# Predation threats for a 24-h period activated the extension of axons in the brains of *Xenopus* tadpoles

**DOI:** 10.1038/s41598-020-67975-7

**Published:** 2020-07-16

**Authors:** Tsukasa Mori, Yoichiro Kitani, Den Hatakeyama, Kazumasa Machida, Naoko Goto-Inoue, Satoshi Hayakawa, Naoyuki Yamamoto, Keiko Kashiwagi, Akihiko Kashiwagi

**Affiliations:** 1 Department of Marine Science and Resources, College of Bioresource Sciences, Nihon University, Kameino 1866, Fujisawa, 252-0880 Japan; 2 Institute of Nature and Environmental Technology, Kanazawa University, Kanazawa, Japan; 3 Department of Pathology and Microbiology, School of Medicine, Nihon University, Tokyo, Japan; 4 Department of Animal Sciences, Graduate School of Bioagricultural Sciences, Nagoya University, Nagoya, Japan; 5 Amphibian Research Center (Building M), Hiroshima University, Hiroshima, Japan

**Keywords:** Molecular ecology, Evolution, Molecular biology

## Abstract

The threat of predation is a driving force in the evolution of animals. We have previously reported that *Xenopus laevis* enhanced their tail muscles and increased their swimming speeds in the presence of Japanese larval salamander predators. Herein, we investigated the induced gene expression changes in the brains of tadpoles under the threat of predation using 3′-tag digital gene expression profiling. We found that many muscle genes were expressed after 24 h of exposure to predation. Ingenuity pathway analysis further showed that after 24 h of a predation threat, various signal transduction genes were stimulated, such as those affecting the actin cytoskeleton and *CREB* pathways, and that these might increase microtubule dynamics, axonogenesis, cognition, and memory. To verify the increase in microtubule dynamics, DiI was inserted through the tadpole nostrils. Extension of the axons was clearly observed from the nostril to the diencephalon and was significantly increased (*P* ≤ 0.0001) after 24 h of exposure to predation, compared with that of the control. The dynamic changes in the signal transductions appeared to bring about new connections in the neural networks, as suggested by the microtubule dynamics. These connections may result in improved memory and cognition abilities, and subsequently increase survivability.

## Introduction

Prey–predator interactions are one of the greatest driving forces of animal evolution, and lead to phenotypic plasticity, i.e., the ability to produce different phenotypes under different environmental conditions^1–4^. The phenotypic plasticity induced by a specific predator is obtained after numerous generations of prey–predator interactions. Encountering an unknown predator, due to environmental changes, might initiate an evolutionary adaptation in the prey, and thus reveal a new model for the initial adaptations that occur in these interactions. Furthermore, it may also shed light on the initial evolutionary processes that occur when adapting to a new predator, which have previously been poorly investigated in ecological scenarios using anuran tadpoles^5^, frogs^6^, and marine bivalves^7^.

Tadpoles can detect predators using both visual and chemical cues^8–10^ and not only modify their behavior to decrease predation risk^11,12^, but also alter their phenotype, resulting in higher survival rates^13,14^. Anuran tadpoles have been extensively studied as a model for phenotypic plasticity. One of their inducible changes is to increase both the height and length of their tails in the presence of a native predation threat, such as the dragonfly larvae^10,15,16^. Japanese predators such as the larval salamander *Hynobius lichenatus*, have also been found to elicit a predation threat response in *X. laevis* tadpoles, resulting in significant enhancements to their tail muscles and decreases in their relative ventral fin heights, leading to significantly higher than average swimming speeds^17^. In this investigation, as the tadpoles were directly preyed upon by the predators, they may be responding to salamander kairomones, visual cues, or conspecific alarm cues released by the damaged tadpoles.

Predation stress causes an increase in the concentration of corticosterone in tadpoles by activating the neuroendocrine stress axis^18^. This indicates that tadpole adaptations against predator involves brain functions, as has previously been reported for other mammals^19,20^. Furthermore, tadpoles have adopted various avoidance strategies to decrease their predation risks (as described above), like modifications to their central nervous systems. At present, there have only been a few previous reports regarding the changes to the brain transcriptome, as a consequence of predation threats^21–25^. However, it is difficult to fully understand the changes to the brain from the expression patterns of only a few genes. Therefore, we have also investigated the changes in the signal transduction pathways of the whole brain, using the transcriptome. Research into the survival adaptations in evolutionary scenarios against unfamiliar predators, using signal transduction analysis in the brain, has not previously been widely conducted.

*X. laevis* tadpoles alter their morphology and behavior to decrease their risk of predation from unfamiliar Japanese predators. During the initial period of the predation threat from Japanese larval salamanders, the tadpoles were found to move away from the predator and become motionless, however this avoidance behavior decreased if the predation threat went on for a period of 10 days. We have postulated that these changes might be associated with alterations in the signal transduction pathways of their brains. In this study we aimed to investigate two problems: first, which signal transduction pathways are altered in the *X. laevis* tadpole brains immediately after acute and chronic predation exposures, and in a post exposure period; and, second, if the adaptations in response to the predation threat occurred over short periods of time, as signal transduction pathways are known to be directly involved with such adaptations.

## Results

### Identification of genes expressed in the brain using 3′-tag digital gene expression profiling

**Figure 1:**
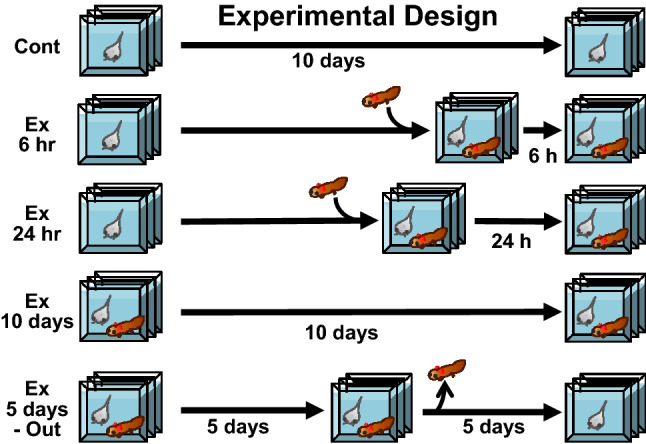
Experimental design for inducing predation in *Xenopus* tadpoles. *Xenopus* tadpoles were assigned to 5 different treatment groups. The control group (Cont) was not exposed to the salamander predator during the experiment (10 days). Tadpoles in the 6 h and 24 h groups were exposed to a salamander larva for 6 or 24 h before sampling, respectively. The 10-day group was exposed to a salamander larva for 10 full days, while the 5-days out group was exposed to a salamander larva for 5 days, to induce the predation threat, then the salamander was removed, and the tadpoles were kept for a further 5 days without the predation threat.

**Figure 2:**
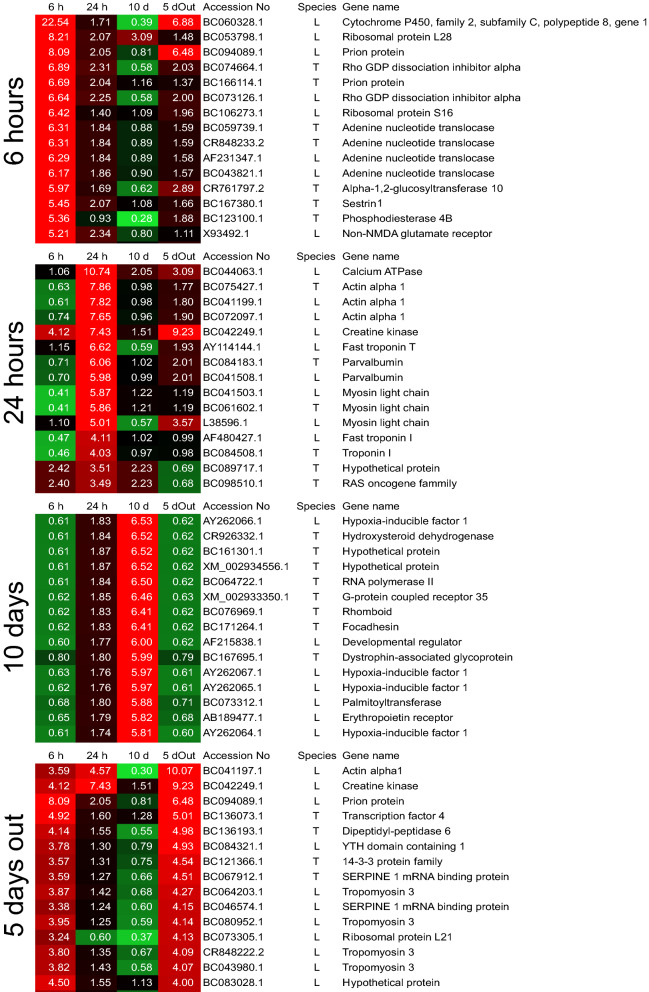
Heat map using 3′-tag digital gene expression profiles from *Xenopus laevis* whole brain tissues. Gene expression profiling was performed using the top 15 genes that were expressed by over 1,000 tags from each of the experimental groups described in Fig. 1. The numbers on the heat map indicate the total number of tags expressed for each gene in the experimental and control groups. Accession No: gene number obtained from the NCBI database. Species: “L” indicates *Xenopus laevis*, and “T” indicates *Xenopus tropicalis*. The heat map colors and numbers indicate the fold changes in gene expression compared with that of the control.

In this experiment, four experimental conditions and an unexposed control were set up as shown in Fig. 1, and the genes expressed in the tadpole brains after ten days were profiled. Approximately 22 to 25 million tags were read, and 41 to 43 thousand different types were hit by the Xenopus blast X. The top 15 genes expressed in the brain owing to the predation threat, in comparison with the control, are listed in Fig. 2. These genes were selected as the number of their expressed tags was greater than 1,000, which indicates that they predict major physiological reactions in the brain.

In the 6-h treatment group, cytochrome P450, the most up-regulated gene, a protein prion, and a non-N-methyl-D-aspartate (NMDA) glutamate gene (which encodes a kainate receptor), had higher expression levels than in the control (Fig. 2). In the 24-h treatment group, a calcium ATPase gene (ATPase, Ca^2+^ transporting, cardiac muscle, slow twitch 2) showed an over tenfold higher expression level than in the control. Surprisingly, most of the expressed genes in the brain after the 24-h predation exposure were muscle-related, such as actin, troponin, and myosin. They showed increased expression compared to the control, and their highest expression levels were about 8 times greater than those of the control. These muscle-related genes were downregulated in the 6-h group but increased in the 5-day-out group. In the 10-day group, the hypoxia-inducible factor gene showed the largest increase (over sixfold) in expression, and it also increased with the time of the exposure, from 6 h to 10 days. In the 5-day-out group, the hypoxia-inducible factor gene fell to the level of expression seen in the 6-h group. Gene expression profiles in the 5-day-out group showed similar tendencies to those of the 6-h group; the gene with the highest expression was actin.

### Pathway analysis of genes expressed in the brain after the threat of predation

**Figure 3:**
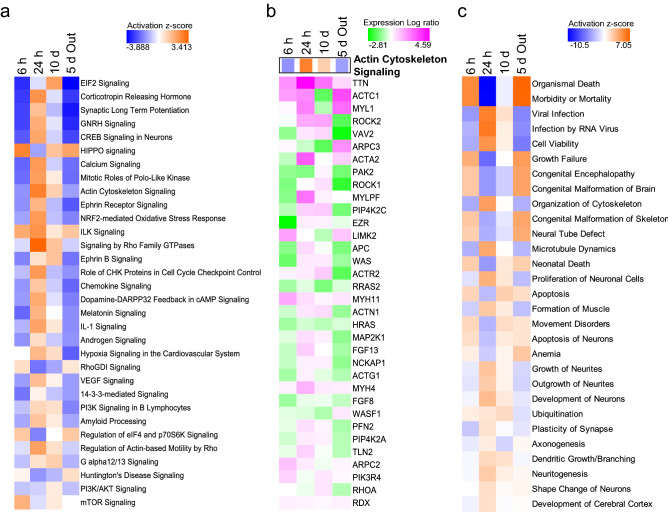
Ingenuity pathway analysis (IPA) of the expressed genes induced in the *Xenopus laevis* whole brain by the threat of predation. Heat map of the altered signal transduction pathways in each treatment group. The number of expressed genes after 6 h (6 h of exposure to a predation threat) were divided by those of the Cont (no exposure to a predation threat) and used to predict the altered signal transduction pathways. 24 h (24 h exposure to a predation threat), 10 days (10 days exposure to a predation threat), and 5 days out (5 days exposure to a predation threat, followed by five days of no threat) also indicate the altered signal transduction pathways compared with that of the Cont, respectively. **(a)** Heat map of the genes expressed in the actin cytoskeleton signaling. Genes involved in actin cytoskeleton signaling were obtained from the IPA, and the levels of expressed genes in each treatment group were compared with that of the Cont. Panels surrounded by the black rectangles, indicate the degree of actin cytoskeleton signaling from **(a)**. The gene symbols and corresponding Entrez Gene Names in **(b)** are given in Supplementary Table 4. **(b)** Disease and function predicted by the analysis of the total signal transduction pathways. The prediction was performed by IPA using the expressed genes compared with those of the control. The displayed diseases and functions are restricted to the top 29 results.

To understand the dynamism in the brains of the tadpoles that were faced with the predation threat, their signal transduction systems were analyzed. In total, 1996 genes were subjected to an IPA pathway analysis using the corresponding zebrafish genes and their signal transduction patterns were analyzed. Signal transduction pathways showing up- or downregulated expression patterns were determined from their z-scores (Fig. 3a; all signal transduction pathways that were altered are shown in Supplementary Table 1). The top 10 up- and downregulated genes are listed in Supplementary Table 2 and were determined from the signal transduction pathway analysis with IPA. These data included the expression of genes with less than 1,000 tags, and the most expressed genes, which were different from those given in Fig. 2. However, similar patterns of change in the gene names in the Supplementary Table 2 and signal transduction pathways were observed in both the 6 h and 5-day-out groups (Fig. 3a). Many of the genes involved in these pathways, such as *EIF2* and corticotrophin releasing hormone, were downregulated in both the 6 h and 5-day-out treatments. By contrast, only 11 signal transduction genes, such as *HIPPO*, *ILK*, *RhoGDI*, *eIF4*, *p70S6K*, and Huntington’s disease, were upregulated in both the 6-h and 5-day-out treatments. However, this was not the case for the *mTOR*, *ATM*, cell cycle, or remodeling of epithelial adherens junction signaling genes (Supplementary Table 1). Various signal transduction pathways, such as those for the actin cytoskeleton and CREB signaling, were increased with the 24-h treatment (Fig. 3a). Upregulation of actin cytoskeleton signaling induced the expression of various muscle related genes as described earlier (Fig. 3b). However, many of these upregulated signal transduction pathways were downregulated in the 10-day treatment, and only four signaling pathways (*EIF2*, *IGF-I*, *AMPK*, and *EGF* signaling) were upregulated after 10 days of treatment (See Supplementary Table 1). Selected diseases and functions were also predicted to vary among the treatment groups (Fig. 3c; predicted diseases and functions are shown in Supplementary Table 3). Increases in organismal death, morbidity or mortality, growth failure, and apoptosis and apoptosis of neurons, were predicted for the 6-h group.

In contrast, increased expression was predicted in the 24-h group for the organization of the cytoskeleton, microtubule dynamics, proliferation of neuronal cells, and axonogenesis. In the 10-day group, apoptosis, movement disorders, ubiquitination, and various cancer related events were predicted to increase (Fig. 3c and Supplementary Table 3). The 5-day-out group showed changes to various disease related events, despite the removal of the predator before sampling; this effect is reminiscent of Post Traumatic Stress Disorder (PTSD).

### Prediction of changes in downstream signal transduction pathways

**Figure 4:**
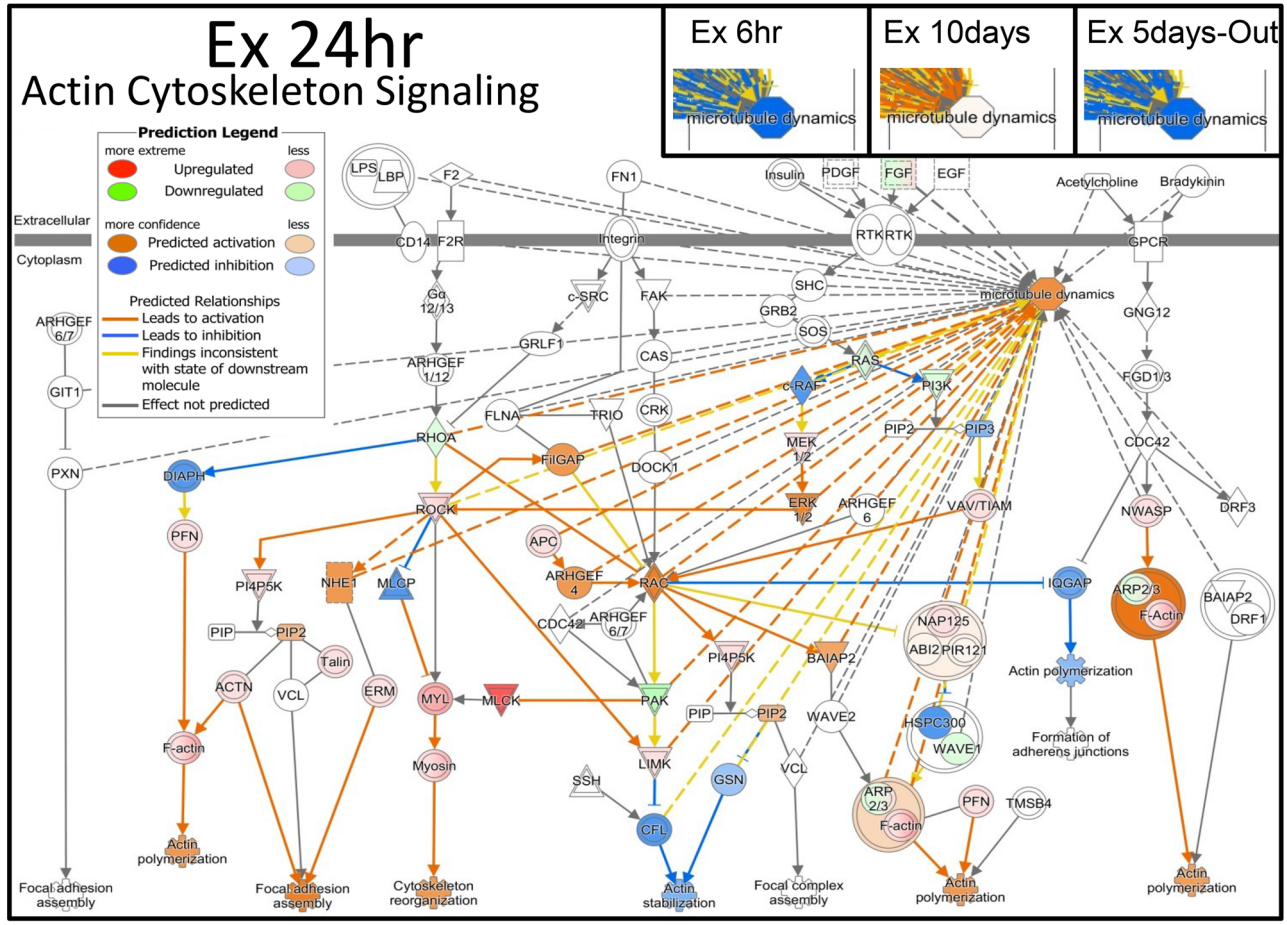
Ingenuity pathway analysis (IPA) of actin cytoskeleton signaling in the *Xenopus laevis* whole brain after exposure to the predatory salamander larva, *Hynobius lichenatus*. Actin cytoskeleton signaling after 24 h of predation (24 h group). The microtubule dynamics shown in the boxes labeled Ex6hr (after 6 h of predation), Ex10days (after 10 h of predation), and Ex5days-Out (after 5 days of predation, followed by 5 days with no predation) in the top right corner, were predicted from the ac**t**in cytoskeleton signaling. The microtubule dynamics in Ex24hr were predicted to be up regulated in the signal transduction map. Blue indicates downregulation and orange indicates upregulation of the microtubule dynamics, compared with the control. The predictions were performed using two statistical measures, an overlapping p‐value, and an activation *z-*score, that were computed for each potential transcriptional regulator. This figure was created using Ingenuity pathway analysis (IPA) version 2.1, QIAGEN; (https://www.qiagenbioinformatics.com/products/ingenuitypathway-analysis).

**Figure 5:**
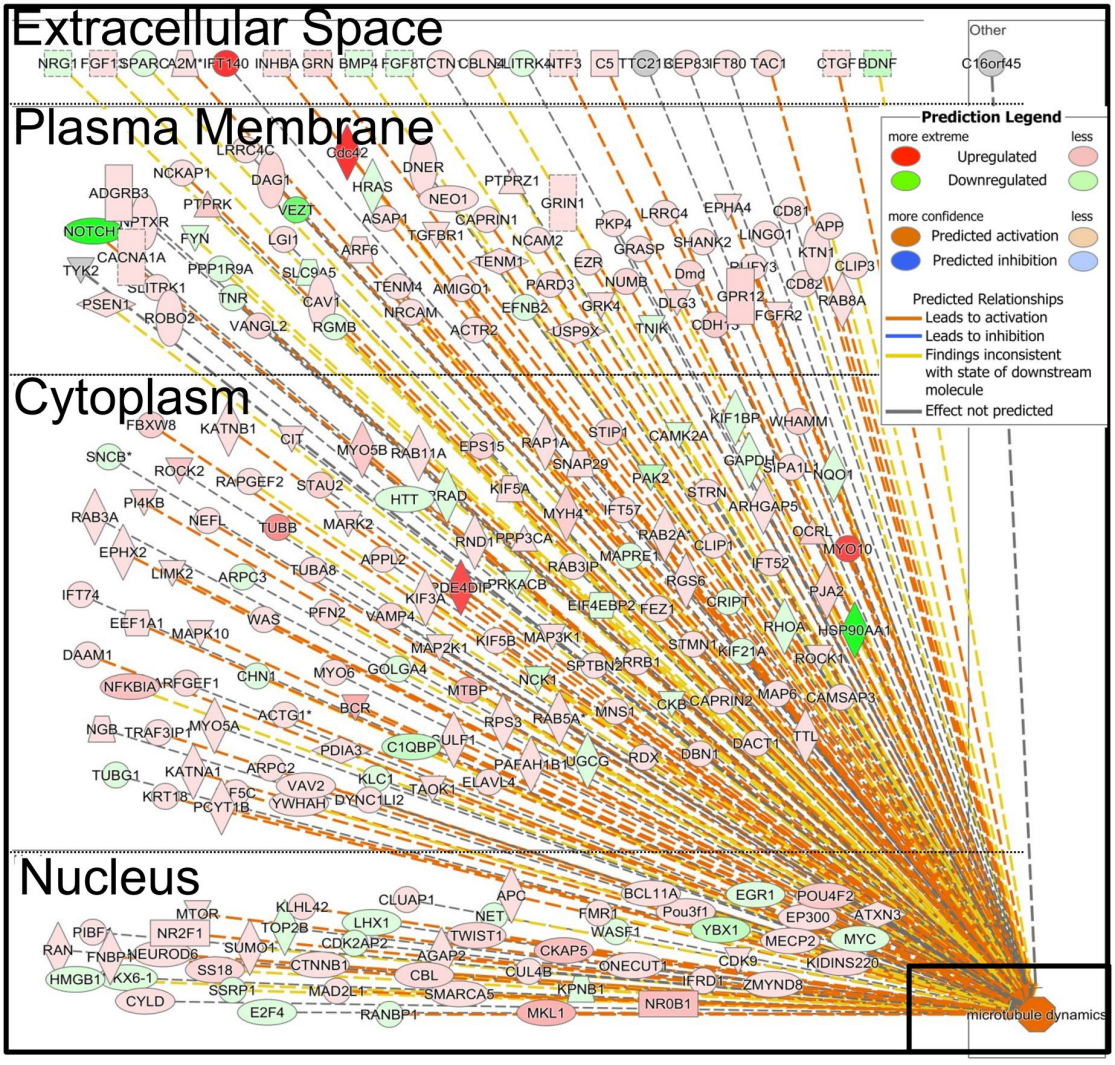
All genes involved in the microtubule dynamics in *Xenopus laevis*, as determined by RNA-seq after 24 h of exposure to a predation threat from the larval salamander *Hynobius lichenatus* (24 h group), in comparison to the control which was not exposed to a predation threat. The black rectangle indicates the microtubule dynamics. This figure was created using Ingenuity pathway analysis (IPA) version 2.1, QIAGEN; (https://www.qiagenbioinformatics.com/products/ingenuitypathway-analysis).

**Figure 6:**
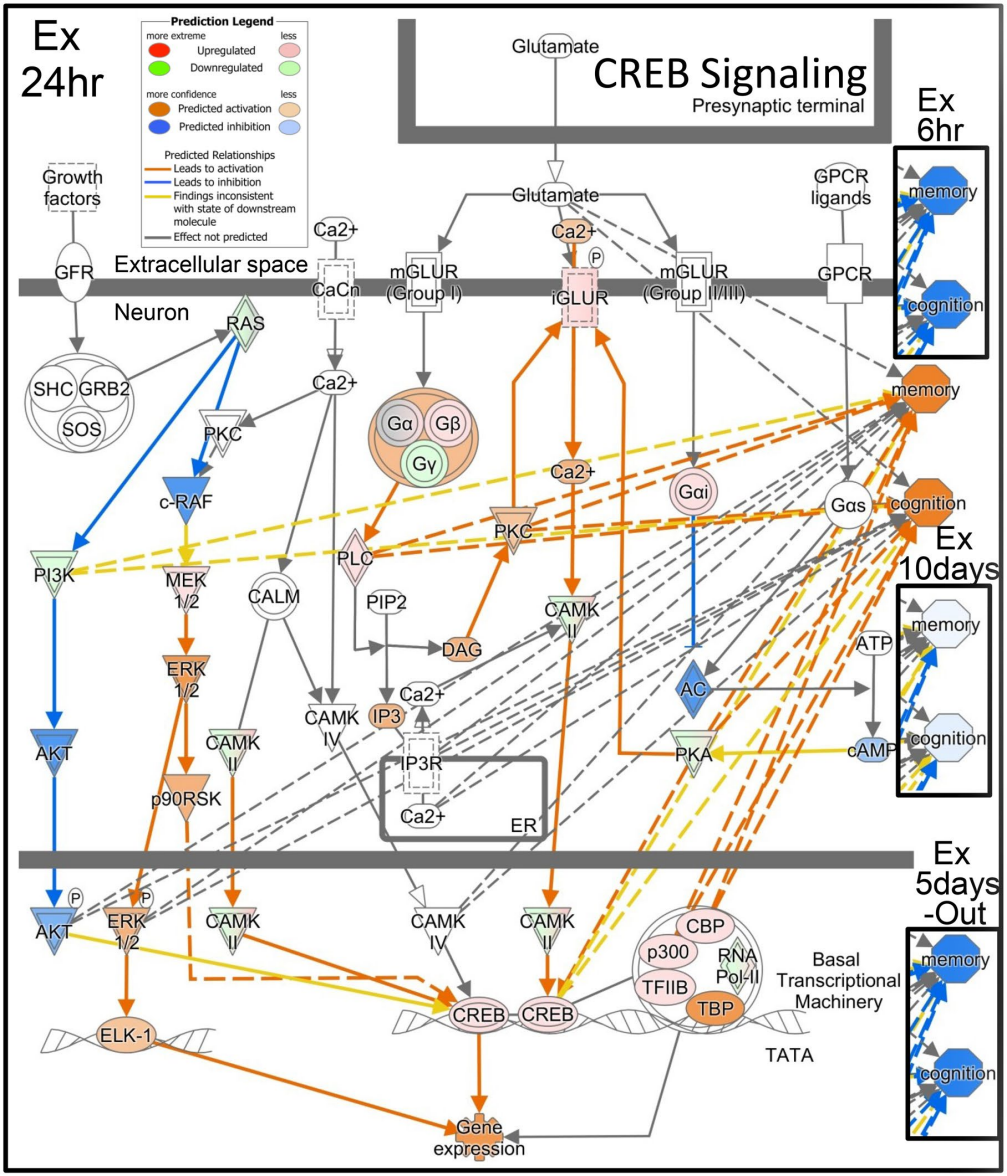
CREB signaling in the *Xenopus laevis* whole brain after exposure to 24 h of a predation threat from the larval salamander, *Hynobius lichenatus*. Changes in the memory and cognition pathways were predicted using the Ingenuity pathway analysis and compared to the controls (not exposed to predation) for the expressed genes in each treatment group. The degree of memory and cognition for Ex6hr (exposed to predation for 6 h), Ex10days (exposed to predation for 10 days), and Ex 5 days-Out (exposed to predation for 5 days, and then 5 days without), are shown in the right-hand side panels.

Various signal transduction pathways showed changes after 24 h of the predation stress (Fig. 3a); two of the most affected downstream pathways were those for the actin cytoskeleton and CREB signaling. Actin cytoskeleton signaling, actin polymerization, focal adhesion assembly, and cytoskeleton reorganization, were all increased in the 24-h group and the network of expressed genes indicated the upregulation of microtubule dynamics (Fig. 4). The predictions indicated the downregulation of the microtubule dynamics in the 6-h and 5-day-out groups; no changes or slight increases were observed in the 10-day group. In total, 108 of the 242 genes involved in the microtubule dynamics showed changes in their expression patterns, consistent with the increased microtubule dynamics in the 24-h group (Fig. 5). CREB signaling is highly involved with memory and cognition and was upregulated in the 24-h group, but downregulated in the 6-h, 10 day, and 5-day-out groups (Fig. 6).

### In situ-hybridization

**Figure 7:**
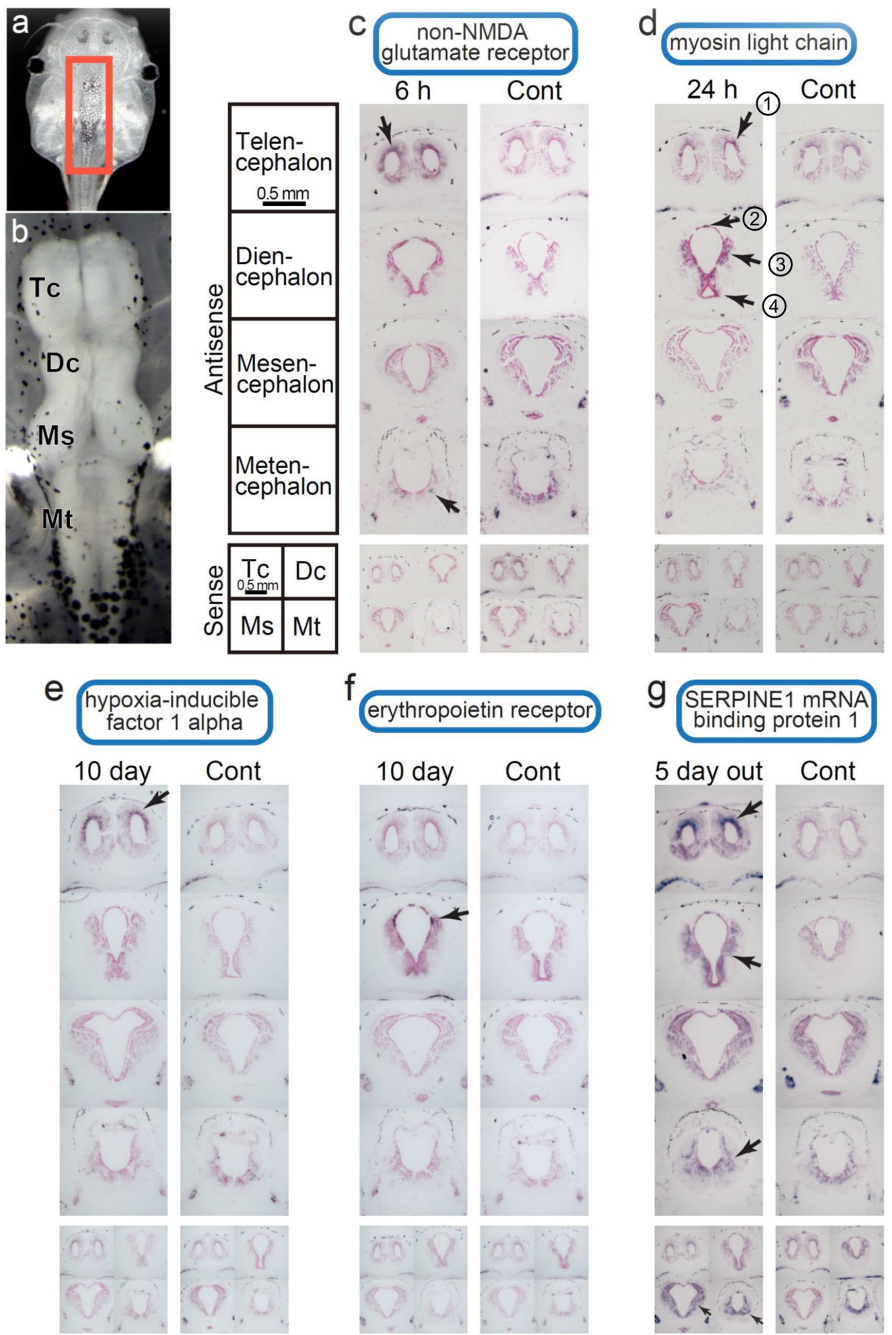
In situ-hybridization in the *Xenopus lavies* brain, using probes for selected genes. *Xenopus laevis* tadpoles sampled from Ex 6 hr (6 h exposure to predation), Ex 24 hr (24 h exposure to predations), Ex 10 days (10 days exposure to predation), and Ex 5 days-Out (5 days exposure to predation and then five days without) were used for in situ-hybridization using selected genes. **(a)** The red rectangle indicates the region of the brain used in this experiment. **(b)** Magnified image of the brain and the names of the brain regions. Tc, Dc, Ms, and Mt indicate the telencephalon, diencephalon, mesencephalon, and metencephalon, respectively. Antisense and sense probes were hybridized to the brain. In situ-hybridization results using anti-sense and sense probe for non-NMDA glutamate receptor **(c)**, myosin light chain **(d)**, hypoxia-inducible factor 1 alpha **(e)**, erythropoietin receptor **(f)**, and serpin 1 mRNA binding protein 1 **(g)**. ① corresponds to the mammalian isocortex; ② indicates a ventral region of the posterior commissure, which may correspond to the subcommissural organ; ③ and ④ indicate the thalamus and hypothalamus in the diencephalon, respectively.

Coronal sections were cut from the tadpoles (Fig. 7a; the red rectangle indicates the brain used in this experiment) and Fig. 7b shows the magnified image of the brain and the brain regions used in these experiments. The quality of RNA preservation was checked by in situ hybridization using a selection of key genes from each predator treatment in the 3′-tag digital gene expression profiling experiment. An anti-sense probe for the non-*NMDA* glutamate receptor (kainate receptor) gave a strong signal in relatively broad areas of the telencephalon, and a weak signal in the metencephalon of the tadpoles from the 6-h treatment group (Fig. 7c). In contrast, no signals were obtained in these regions when the adjacent sections from the same tadpole were examined with a sense-RNA probe. Control tadpoles showed very weak signals in the telencephalon and somewhat stronger signals in the metencephalon, compared to the 6-h group, using the anti-sense RNA probe for the kainite receptor (Fig. 7c). Signal intensities of the sense RNA probe in the telencephalon, diencephalon, and metencephalon were far weaker in the control tadpoles than with the anti-sense probe, and they showed different staining patterns.

In the 24-h group, a myosin anti-sense RNA probe was used to detect muscle related genes expressed in the brain and hybridized to the dorsal pallium shown in Fig. 7d, ①, which corresponds to the mammalian isocortex. In the diencephalon, clear signals were obtained in a ventral region of the posterior commissure (Fig. 7d, ②), which may correspond to the subcommissural organ. Signals were also recognized in the thalamus and hypothalamus in the diencephalon (see Fig. 7d, ③ and ④, respectively), whereas no signals were found in the corresponding regions of the controls. Therefore, myosin gene expression differed between the 24-h group and the controls (Fig. 7d).

In the tadpoles that received the 10-day treatments, an anti-sense Hif1α probe showed clear signals in the region of the telencephalon, which eventually forms the medial, dorsal, and lateral pallia (Fig. 7e), while only weak signals were observed in the controls. An anti-sense erythropoietin receptor probe showed clear signals in the dorsal part of the thalamus (Fig. 7f), and the signal intensities were stronger than those in the controls. No signals were observed with Hif1α and the erythropoietin receptor sense probes.

In the 5-day-out group, a serpin 1 mRNA binding protein (SERBP1) anti-sense probe gave a strong signal in the telencephalon and weak signals in the diencephalon and metencephalon (Fig. 7g); weaker signals were found in the controls. Interestingly, the sense probe hybridized to the mesencephalon and metencephalon. However, the signal intensities and patterns differed from those obtained with the anti-sense probe.

### Evaluation of microtubule dynamics using DiI

**Figure 8:**
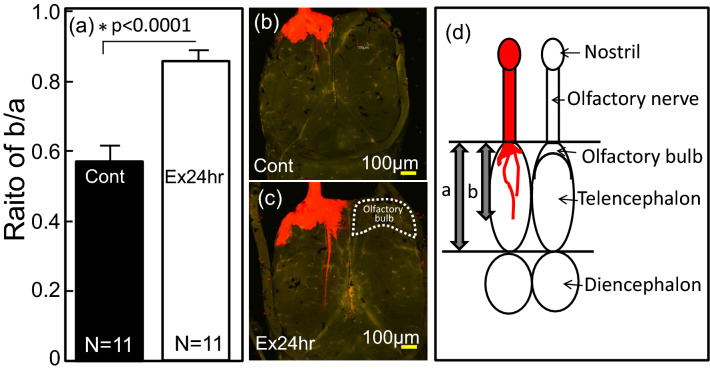
Extension of the axons in the brains of *Xenopus laevis* tadpoles exposed to the Japanese predatory larval salamanders, *Hynobius lichenatus* for 24 h. **(a)** Ratio of the extended axons per telencephalon compared with the control (Cont.; not exposed to Japanese predators) was calculated as b/a (a: length of telencephalon; b: length of the extended axons) in the brain. Statistical analysis (*t*-test) on the data were performed with SPSS. **(b)** Tadpoles not exposed to a predator **(c)** Tadpoles exposed to Japanese predator for 24 h. **(d)** Schematic profile of a tadpole’s brain stained by DiI.

To evaluate how the activation of the actin cytoskeleton signaling affected the microtubule dynamics in the tadpoles exposed to 24 h of predation, DiI was inserted into the nostrils of the tadpoles and the axon extensions in the tadpole brains examined (Fig. 8). DiI was diffused in the neuron, and the ratio of the length against telencephalon was significantly increased (*P* ≤ 0.0001) (Fig. 8a). The pattern of extension in the neural axons of the tadpoles exposed to the predation threat (Fig. 8c) was like that in the other samples, and the stained area of the olfactory bulb increased, compared to that of the control. The extension of the axon was directed towards the diencephalon (Fig. 8d).

## Discussion

The gene expression profile of the tadpole brain (Fig. 2) is extremely useful when deducing the response of a tadpole to an unknown predator. After 6 h of exposure to the predation threat, the cytochrome P450 enzymes (CYPs) showed approximately 23-fold greater expression, compared with that of the controls. These enzymes are part of a well-known superfamily of hemeproteins, functioning as the terminal oxidases of drug metabolizing enzymes, i.e. enzymes that catalyze the biotransformation of drugs and xenobiotics, and are involved with the metabolism of endogenous compounds that generate damaging toxic metabolites and cause oxygen stress^26^. CYPs have tissue specific expression, and metabolize compounds acting as drugs neurotoxins, neurotransmitters, and neurosteroids in the brain^27–29^. Furthermore, the processes of the CYPs produce oxidants as byproducts^30^. This means that tadpole brains perform detoxification, and this corresponds with the observed result that the greatest increase in organismal death was after 6 h of exposure to the predation stress (Fig. 3c).

These predictions are supported by the results showing a greater expression of prions in the tadpoles brain, as prions are also known to be responses to oxidative stress^31^ or apoptosis^32^. These reactive oxygen species (ROS) and the resulting oxidative stresses, play an important role in apoptosis^33^. At the same time, important tissues and cells must be protected from the generated ROS. Adenine nucleotide translocase (ANT) is the protein in the mitochondrial membrane^34^, and overexpression of the enzyme compensates impaired ANT activity under oxygen-restricted conditions. Increases of ANT reduces ROS production and oxidative stress, stabilizes mitochondrial integrity, and increases survival^35^. Phosphodiesterase 4B is a marker of alcohol-induced peripheral endotoxemia mediated neuro-inflammation^36^, and is involved with inflammatory responses in the brain through inflammasomes, such as caspase-1^37^. Expression of caspase 1 in the 6-h treatment group (tag counts: 1,600) was about two times greater than in that of the control (tag counts: 834). This indicates that the shock of the predation threat for the initial 6 h, resulted in oxidative stress, inflammatory responses, and apoptosis in the tadpole brain. Furthermore, synaptic long-term potentiation signaling was downregulated in the 6-h group, although mTOR signaling was upregulated (Fig. 3a). mTOR is a central integrator of various signaling pathways, such as different forms of synaptic plasticity and long-term memory formation, and mediates the consolidation of fear memories^38–40^. The consolidation of fear memories is required for the elevation of phosphorylated downstream targets of mTOR (p70s6k and 4E-BPs) in the hippocampus, amygdala, and prefrontal cortex^41^. However, mTOR was upregulated, regardless of the downregulation of the synaptic plasticity (Fig. 3c) or synaptic long-term potentiation (Fig. 3a). This contradiction may suggest that the consolidation of fear memories occurs in a limited area of the brain after 6 h, whereas plasticity of the synapses (Fig. 3c), which are involved in memory consolidation, did not occur at this time, as indicated by the up- and downregulated genes after 6 h of exposure.

Eukaryotic initiation factor 2 (eIF2) is a master regulator of protein synthesis and plays a major role in protein translation initiation. eIF2α phosphorylation is induced by several stress conditions and reduces protein synthesis while allowing preferential translation of some mRNAs^42^. As described above, various signal transduction pathways might be downregulated following the inhibition of protein synthesis due to the downregulation of eIF2 after 6 h of predation exposure.

However, after 24 h of exposure to the Japanese predator, dynamic changes to the signal transduction pathways evidently occurred in the brain for survival. We focused our attention on muscle-related gene expression, such as that of actin and myosin (Fig. 2), which are part of the actin cytoskeleton signaling network (Fig. 3b and Supplementary Table 4). Actin is one of the most abundant proteins in neurons, and filamentous actin forms bundles and networks that are components of the actin cytoskeleton. Microtubules form important cytoskeletal structures to maintain neuronal polarity, regulate neuronal morphology, and transport scaffold signaling molecules^43^.

Within a neuronal cell, microtubules have variable lengths and can be both stable and dynamic structures, that can quickly adapt to changes in the environment^43,44^. The stability of microtubules is necessary for axon formation, for maintaining the identity of axons, and for regulating the dynamics of dendritic spines^45,46^; they are regulated by GTPases of the Rho family^47^. As shown in Fig. 3a, the Rho family GTPase and actin cytoskeleton signaling were upregulated; therefore, after 24 h of exposure to a predator, increased microtubule dynamics stimulated neurite growth, axonogenesis, and the development of neurons.

The prediction of microtubule dynamics by the activation of actin cytoskeleton signaling, was evaluated directly with the insertion of DiI in the tadpole nostril after 24 h of exposure to predation (Fig. 8). The stained area of the olfactory bulb of the tadpoles exposed to the predation threat (Fig. 8c) seemed to spread, when compared to that of the control (Fig. 8b). This may be related to the expression results of the myosin mRNA, as the probe for microtubule dynamics. It was detected in the dorsal pallium (Fig. 7d), and the lateral, medial, and dorsal pallia of fish that have been proposed to be homologous to the mammalian olfactory (piriform) cortex, hippocampus, and isocortex, respectively^48^. The presumed medial and lateral pallial homologues in fish are functionally different and are necessary for spatial learning and emotional memory, respectively^49^. The dorsal pallium has been implicated in trace avoidance conditioning but not in non-trace avoidance conditioning in fish; the dorsal pallium is principally related to short-term memory processes^48^. Therefore, tadpoles may try to increase trace avoidance and short-term memory processes. In mammals, there are some reports that aversive stimuli alter the olfactory bulb^50^. Olfactory sensory neurons are the first cells in the olfactory system, and axon projection happened within the olfactory bulbs when mice physically encountered odor molecules in their noses^51^. However, there are no reports showing axon extension in the olfactory bulb, or in the telencephalon, after only a short period of prey-predator interactions.

In this report, it was clearly observed that axon projections were from the olfactory bulb to the diencephalon (Fig. 8a, c). As shown in the myosin RNA that was identified in the presumed subcommissural organ, a periventricular organ whose functions remain unknown, the myosin RNA expressions were also found in the thalamus that relays sensory information to the telencephalon^52,53^ and in the hypothalamus, which is involved in endocrine control and autonomic functions^54^. These results may also indicate that tadpoles try to connect axons to the thalamus and hypothalamus to change hormonal conditions during a 24-h predation threat. This might relate to the 3.67- and 2.34-times greater expression of growth hormone 1 and prolactin mRNA, respectively, compared with the control at 24 h. These data also indicate that tadpoles highly respond to salamander kairomones and conspecific alarm cues released by the damaged tadpoles.

Furthermore, ephrin receptor signaling was upregulated in tadpoles exposed to a predator for 24 h. The Eph receptors and their ephrin ligands are key conserved regulators of axon guidance^55^. Here, we predicted the upregulation of Erk1/2 with IPA analysis (it was up regulated only at 24 h) downstream of the Eph receptor signaling (Fig. 3a), which suggested the upregulation of axon guidance, as has been reported previously^56^. The upregulation of ephrin B signaling was also present in the 24-h treatment group (Fig. 3a). The functions of the ephrin B receptor are not limited to the control of the actin cytoskeleton in dendritic spines, as shown by ephrin receptor signaling, but are also involved in the formation of functional synaptic specializations through the regulation of protein transport by glutamate receptors^57^. There is an interesting report that shows that reducing EphB2 synthesis in the infralimbic cortex accelerates fear extinction learning in adolescent rats^58^. In our data, both EphB and EphB receptors, were upregulated after exposure to a predator for 6 h; however, they were progressively downregulated after 24 h and 10 days of exposure. These results indicate that the tadpole fear response was initiated after 6 h in the presence of the predator, and that fear extinction occurred with increased time in its presence.

CREB signaling in neurons was also upregulated after 24 h of predation (Fig. 3). CREB is a transcription factor and is activated by phosphorylation in response to external stimuli^59^. Numerous reports indicate that CREB-dependent gene expression is essential for long-term memory, cognition, and plasticity in the nervous system. However, in the 6-h group, CREB and its basal transcriptional machinery, such as TBP (TATA box binding protein), TFIIB (general transcriptional factor IIB), CBP (CREB binding protein), and p300 (E1A binding protein p300), were downregulated. Interestingly, TFIIB was upregulated in the 10-day group, suggesting a difference in the memory and cognition between the 6 h and 10-day groups (Fig. 6).

A higher expression level of the non-NMDA glutamate receptor (kainate glutamate receptor) gene was detected using the RNA-seq and in situ hybridization and showed that it was present around the dorsal pallium of the tadpoles after exposure to a predator for 6 h (Fig. 7c). Ionotropic glutamate receptors are known to be involved in synaptic transmissions in the brain, and are classified into three types: NMDA, α-amino-3-hydroxyl-5-methyl-4-isoxazole- propionate (AMPA), and kainate; the latter two receptors are included in the non-NMDA glutamate receptor^60^. This gene is involved in glutamate receptor signaling which mediates neuron depolarization, synaptic plasticity, long-term potentiation, and learning though CREB. We found that the kainate types of the glutamate receptors were upregulated in the 6 h and 24-h groups, and that the NMDA receptor was also upregulated in the latter. The AMPA receptor is a glutamate-gated cation channel that mediates fast excitatory neurotransmission and synaptic plasticity; we found that GRIK (glutamate receptor, ionotropic, kainate) was up-regulated faster than GRIA (glutamate receptor ionotropic AMPA glutamate receptor) in the 6 h group^61^. However, all these receptors were downregulated in the 10-day group. Upregulation of the kainate receptor in the 6 h group might indicate initial preparations for the synapse elongation that enhances short-term memory processes^48^.

In the 10-day group, exposure to the continual predation threat for this period might have induced the greatest response, as expression of the hypoxia-inducible factor 1 and erythropoietin receptor gene were at their highest (Fig. 2). Hypoxia-inducible factors mediate cell transcription responses to hypoxia^62^. Hypoxia-inducible factor 1 is increased in cells exposed to low oxygen and binds to the promoter of the erythropoietin gene^63^. Our results therefore suggest that the tadpoles suffered oxygen deficiency during the 10 days of predation stress, which caused the increased production of erythrocytes, owing to chronic stress. The deficiency of oxygen may be related to changes in the tadpole behavior, such as the decreased avoidance response to the larval salamanders after exposure to the predation threat for 10 days.

Thus, in the initial 24 h, the tadpoles exposed to the unfamiliar Japanese predators stimulated various signal transductions, by synthesizing various proteins to search for the best way to survive. This strategy requires a great energy input from the tadpole, when generally, saving energy in desperate situations to improve the chances of survival, is the priority for animals. In these experiments, *Xenopus* tadpoles searched various signal transduction mechanisms, to find a suitable defense against an unfamiliar Japanese predator in a short period of time, at the expense of a great amount of energy. Therefore, they changed their brain neural networks through the activation of microtubule dynamics, which predicted increases of memory and cognition by stimulating the CREB signaling pathways. However, the 5-day-out group showed defects in cellular function, such as neural tube defects, neonatal death, and growth failure, like the effects observed with PTSD after exposure to a predator. Analysis of the dynamic changes in signal transductions and diseases and function using IPA in the tadpole brain after experiencing fear stress, will be valuable to better understand the effects of stress in both humans and experimental animals.

## Materials and methods

### Experimental animals and design

Larval *Hynobius lichenatus* salamanders were purchased from a commercial dealer (Toshin Co. Tsukuba, Japan), reared in aquaria (25 L), and fed *Tubifex *ad libitum until they were ~ 4 cm in body length. *Xenopus laevis* tadpoles (J strain, Stage 51) were purchased from a commercial dealer (Watanabe Zoushyoku, Hyougo, Japan) and fed boiled and pureed green peas (2 g) every two days during the experimental period. The tap water used was first treated with activated charcoal (Tsurumi Coal. Yokohama, Japan) for 24 h. The experiment was conducted in a laboratory at 20 °C, using a natural day/night regime (~ 14/10 h). The experiments were performed using aquaria (2.5 L; 25 × 10 cm surface area; 10 cm height), containing treated tap water (2 L). Fifty similarly sized tadpoles were chosen randomly from a holding tank (20 L) and placed in each aquarium. The water in all aquaria was changed every second day throughout the experiment.

Four experimental conditions and an unexposed control were set up: the predator treatments were for 6 h, 24 h, or 10 days followed by examination, or for 5 days but examined 5 days after the end of the treatment (5-day-out). There were three replicate aquaria for each treatment group. For the 10 day and 5-day-out groups, there were 6 backup aquaria. All treatment groups were initiated at the same time and maintained for 10 days. The larval salamanders were introduced into the aquariums at the appropriate times, prior to sampling the tadpoles (Fig. 1). All treatment groups were sampled at the end of day 10. During the experiment, to maintain the tadpole population density in the predator treatment groups, the salamanders were replaced daily with randomly selected individuals from a holding tank that contained a readily available supply of *T. tubifex*. Since the survival of the tadpoles was crucial for this experiment, we tried to maintain a survival rate of more than 70% by changing the salamanders. We counted the number of surviving tadpoles in each aquarium daily. For the 10 day and 5-day-out groups, any loss of tadpoles to predation and moribund tadpoles were compensated for by using replacement tadpoles from a backup aquarium, chosen arbitrarily, to maintain a minimum of 50 tadpoles per aquarium in each treatment, to eliminate any possible density effect on the results. The weight of the tadpoles is shown in Supplementary Table 5.

### RNA extraction and 3′-tag digital gene expression profiling for RNA-seq

Ten tadpoles were collected from each aquarium (therefore, 30 tadpoles per treatment group) and used for RNA sequencing (RNA-seq). The head part of the tadpoles was cut and placed in tubes containing RNAlater (Qiagen) and stored overnight at 4 °C. Brain tissues were dissected under the microscope, and only brain tissues were kept at − 80 °C until used for RNA extraction. Total RNA was purified from each sample using an RNeasy Midi Kit according to the manufacturer's protocol. The quality of total RNA was determined by measuring the *A*_260_/*A*_280_ absorbance ratio (between 1.7 and 2.1). Further, the clear band of 18S and 28S ribosomal RNA were checked by using RNA gel electrophoresis, and the RNA showing ribosomal RNA bands ratio was 1:2 were further processed for the RNA-seq. Total RNAs (10 μg) from brain tissues collected from tadpoles in each of the five treatment groups were used to construct libraries for Illumina sequencing using 3′-region tags, according to the protocol described for the Digital Gene Expression-NlaIII Sample Preparation Kit (Illumina, San Diego, CA). Oligo-dT magnetic beads were used for mRNA purification from total RNA, and the first-strand cDNA was synthesized using reverse transcriptase. Then, second-strand cDNA was produced using DNA polymerase and RNaseH and obtained double-stranded cDNAs. They were treated with the NlaIII restriction enzyme, which recognizes the CATG site, and thus sequences of the CATG site remained in the 3′-regions in the cDNA library connected with beads. The sequences of the 3′-regions were ligated to adapter 1, and then digested with EcoP15I from beads. By digested with EcoP15I, fragments of 22 to 24 bp cDNA were obtained because the EcoP15I restriction site is 5′-CAGCAG(N)_25_–3′. These cDNA fragments purified from beads were ligated to adapter 2, and amplified only cDNA ligated to both adapter 1 and 2 by PCR using NEBNext Mutiplex Oligos for Illumina (E7335, New England Biolabs, Ipswich, MA). Further, the PCR products of approximately 85 bp were purified on a PAGE gel and used as libraries. The detail structures of adaptor and primer sequences are given in Supplementary Table 6. Sequence of cDNA was performed using an Illumina GA IIx sequencer. In this analysis, we obtained 38 bp reads from one lane per sample (InfoBio, Tokyo, Japan). The sequence data was deposited in DDBJ BioProject database **(**PRJDB8548). After the sequence, the NlaIII site (CATG) was added to the 5′ end of the cDNA, and removed 6 or more nucleotides at the 3′ end of the adapter 2 from the reads. The analysis is in line with a previously published methodology^64^. Except that this study utilized ExoP15I as the enzyme for cutting off tags instead of MmeI which was used in the previous study. The gene tags with a length of 26 to 28 nucleotides were applied for gene expression analysis. The gene tags were aligned with a reference gene sequence for *Xenopus tropicalis* from the National Center for Biotechnology Information (https://www.ncbi.nlm.nih.gov/nuccore) using BLAST 2.2.23. Gene expression levels were determined similarly to the previous method^64^.

### Pathway analysis of genes selected by IPA analysis

An Ingenuity Pathway Analysis (IPA, Qiagen) was used to identify functional networks of genes expressed in the brain because of predation stress. To perform the IPA analysis, gene IDs obtained from the *Xenopus* species database were changed to IDs corresponding to zebrafish genes using BioMart (https://www.biomart.org/). For *Xenopus* genes that did not identify an ID from the zebrafish database, the genes with the greatest similarity in the latter database were found using tblastx. Gene expression ratio to the control using Tag from RNA-Seq was calculated as: log_2_(Tag countSAMPLE + 1) − log_2_(Tag countCONTROL + 1). IDs were consolidated using the expression value set as “average” and the measurement for resolving duplicates set as “Ex Log Ratio”. A total of 2,821 IDs were obtained, and 2,053 were mapped and signal transduction pathways were identified using IPA with 1,966 genes (eliminated duplicated genes). The activation *z*‐score is used to infer likely activation states of the upstream regulators based on a comparison with a model that assigns random regulation directions (see manual of Ingenuity Upstream Regulator Analysis in IPA). These computational analyses were performed using version IPA 2000-2016 QIAGEN.

### In situ hybridization

Tadpoles were embedded in an Optimal cutting temperature compound (O.C.T) each embedded tadpole was soaked in dry-iced n-hexane, and then 5 μm sections were cut using a Leica CM1950 cryostat (Leica). The sections were attached to Kawamoto method film^65^, and then treated with 4% paraformaldehyde. After proteinase K treatments, in situ hybridization was carried out using the DIG system (Nacalai Tesque), as described previously^66^.

### Microtubule dynamics with DiI

The larval salamander *H. lichenatus* could not be sourced at the time this experiment was conducted, so instead, *Hynobius retardatus*, a salamander of the same species, was used. Eggs of *H. retardatus* were collected from a pond in Hokkaido, Japan, and reared until the experiment^67^. The experimental design that was described previously to examine the 24-h predation threat and control, was used again, and then the tadpoles were fixed with 4% paraformaldehyde and stored at 4 °C, prior to injection with DiI (1,1′-dioctadecyl-3,3,3′,3′-tetramethylindocarbocyanine perchlorate). Three mg of DiI (Aldrich chemistry) were dissolved in DMSO (400 µL), and the pigment was taken up by capillary action. The same amount of dissolved DiI was injected into the left nostril using capillary glass by Femtojet 4i (Eppendorf, Hamburg, Germany). The injection conditions were as follows: pressure, 20 hPa; time, 0.20 s; maintenance pressure, 5 hPa. After injecting the DiI, the nostril was covered with melted gelatin (8% with water w/v) and incubated at 35 °C for 7 days with 2% paraformaldehyde, under dark conditions. Each capillary glass was used only once for each sample, to inject the constant volume of DiI. After incubation, the brain was carefully removed under a stereomicroscope (Leica MZ16) and observed, using constant conditions, by confocal microscopy (Olympus). Pictures were obtained from compressed Z-stack images, analyzed with Image-J (https://imagej.nih.gov/ij/), and then statistical tests (*t*-test) were conducted with SPSS.

### Ethic approval and consent for participation

All animal experiments were conducted by trained personnel in accordance with the guidelines of the Animal Care Committee, Nihon University. Ethical approval for the use of animals was given by the University of Nagoya Research Ethics Committee (#2012041301).

## Supplementary information

Supplementary file1.

## Data Availability

The sequence data are available under accession number of the DDBJ BioProject database (PRJDB8548).

## References

[1] Spitze K (1992). Predator-mediated plasticity of prey life history and morphology: *Chaoborus americanus* predation on *Daphnia pulex*. Am. Nat..

[2] Schoeppner NM, Relyea RA (2005). Damage, digestion, and defence: The roles of alarm cues and kairomones for inducing prey defences. Ecol. Lett..

[3] Relyea RA (2018). Phylogenetic patterns of trait and trait plasticity evolution: Insights from amphibian embryos. Evolution Int. J. Organic Evolut..

[4] Reger J, Lind MI, Robinson MR, Beckerman AP (2018). Predation drives local adaptation of phenotypic plasticity. Nat. Ecol. Evolut..

[5] Nunes AL, Richter-Boix A, Laurila A, Rebelo R (2013). Do anuran larvae respond behaviourally to chemical cues from an invasive crayfish predator? A community-wide study. Oecologia.

[6] Johnston CA, Wilson Rankin EE, Gruner DS (2018). Foraging connections: Patterns of prey use linked to invasive predator diel movement. PLoS ONE.

[7] Hollander J, Bourdeau PE (2016). Evidence of weaker phenotypic plasticity by prey to novel cues from non-native predators. Ecol. Evolut..

[8] Relyea RA (2000). Trait-mediated indirect effects in larval anurans: Reversing competition with the threat of predation. Ecology.

[9] Gallie JA, Mumme RL, Wissinger SA (2001). Experience has no effect on the development of chemosensory recognition of predators by tadpoles of the American toad, *Bufo americanus*. Herpetologica.

[10] McCollum SA, Leimberger JD (1997). Predator-induced morphological changes in an amphibian: Predation by dragonflies affects tadpole shape and color. Oecologia.

[11] Relyea RA (2001). Morphological and behavioral plasticity of larval anurans in response to different predators. Ecology.

[12] Fraker ME (2008). The effect of hunger on the strength and duration of the antipredator behavioral response of green frog (*Rana clamitans*) tadpoles. Behav. Ecol. Sociobiol..

[13] VanBuskirk J, Relyea RA (1998). Selection for phenotypic plasticity in *Rana sylvatica* tadpoles. Biol. J. Linn. Soc..

[14] Kishida O, Nishimura K (2005). Multiple inducible defences against multiple predators in the anuran tadpole, *Rana pirica*. Evolut. Ecol. Res..

[15] Van Buskirk J, McCollum SA, Werner EE (1997). Natural selection for environmentally induced phenotypes in tadpoles. Evolution Int. J. Organic Evolut..

[16] Kishida O, Trussell GC, Mougi A, Nishimura K (2010). Evolutionary ecology of inducible morphological plasticity in predator-prey interaction: Toward the practical links with population ecology. Popul. Ecol..

[17] Mori T (2017). The constant threat from a non-native predator increases tail muscle and fast-start swimming performance in Xenopus tadpoles. Biol. Open.

[18] Middlemis Maher J, Werner EE, Denver RJ (2013). Stress hormones mediate predator-induced phenotypic plasticity in amphibian tadpoles. Proc. Biol. Sci..

[19] Adamec R, Kent P, Anisman H, Shallow T, Merali Z (1998). Neural plasticity, neuropeptides and anxiety in animals—Implications for understanding and treating affective disorder following traumatic stress in humans. Neurosci. Biobehav. Rev..

[20] Figueiredo HF, Bodie BL, Tauchi M, Dolgas CM, Herman JP (2003). Stress integration after acute and chronic predator stress: Differential activation of central stress circuitry and sensitization of the hypothalamo-pituitary-adrenocortical axis. Endocrinology.

[21] Jongren M, Westander J, Natt D, Jensen P (2010). Brain gene expression in relation to fearfulness in female red junglefowl (*Gallus gallus*). Genes Brain Behav..

[22] Sanogo YO, Hankison S, Band M, Obregon A, Bell AM (2011). Brain transcriptomic response of threespine sticklebacks to cues of a predator. Brain Behav. Evol..

[23] Fraser BA, Weadick CJ, Janowitz I, Rodd FH, Hughes KA (2011). Sequencing and characterization of the guppy (*Poecilia reticulata*) transcriptome. BMC Genomics.

[24] Drew RE (2012). Brain transcriptome variation among behaviorally distinct strains of zebrafish (*Danio rerio*). BMC Genomics.

[25] Cinel SD, Taylor SJ (2019). Prolonged bat call exposure induces a broad transcriptional response in the male fall armyworm (*Spodoptera frugiperda*; Lepidoptera: Noctuidae) brain. Front. Behav. Neurosci..

[26] Miksys S, Tyndale RF (2004). The unique regulation of brain cytochrome P450 2 (CYP2) family enzymes by drugs and genetics. Drug Metab. Rev..

[27] Ekins S, Wrighton SA (1999). The role of CYP2B6 in human xenobiotic metabolism. Drug Metab. Rev..

[28] Hiroi T (2001). Progesterone oxidation by cytochrome P450 2D isoforms in the brain. Endocrinology.

[29] Seliskar M, Rozman D (2007). Mammalian cytochromes P450–importance of tissue specificity. Biochim. Biophys. Acta.

[30] Borkum JM (2016). Migraine triggers and oxidative stress: A narrative review and synthesis. Headache.

[31] Brown DR, Schulz-Schaeffer WJ, Schmidt B, Kretzschmar HA (1997). Prion protein-deficient cells show altered response to oxidative stress due to decreased SOD-1 activity. Exp. Neurol..

[32] Paitel E, Fahraeus R, Checler F (2003). Cellular prion protein sensitizes neurons to apoptotic stimuli through Mdm2-regulated and p53-dependent caspase 3-like activation. J. Biol. Chem..

[33] Kannan K, Jain SK (2000). Oxidative stress and apoptosis. Pathophysiology.

[34] Klingenberg M (2008). The ADP and ATP transport in mitochondria and its carrier. Biochim. Biophys. Acta.

[35] Klumpe I (2016). Transgenic overexpression of adenine nucleotide translocase 1 protects ischemic hearts against oxidative stress. J. Mol. Med. (Berlin, Germany).

[36] Avila DV (2017). Phosphodiesterase 4b expression plays a major role in alcohol-induced neuro-inflammation. Neuropharmacology.

[37] You T (2017). Roflupram, a phosphodiesterase 4 inhibitor, suppresses inflammasome activation through autophagy in microglial cells. ACS Chem. Neurosci..

[38] Glover EM, Ressler KJ, Davis M (2010). Differing effects of systemically administered rapamycin on consolidation and reconsolidation of context vs cued fear memories. Learn. Mem. (Cold Spring Harbor, N.Y.).

[39] Parsons RG, Gafford GM, Helmstetter FJ (2006). Translational control via the mammalian target of rapamycin pathway is critical for the formation and stability of long-term fear memory in amygdala neurons. J. Neurosci..

[40] Slipczuk L (2009). BDNF activates mTOR to regulate GluR1 expression required for memory formation. PLoS ONE.

[41] Fifield K (2015). Time-dependent effects of rapamycin on consolidation of predator stress-induced hyperarousal. Behav. Brain Res..

[42] Sidrauski C (2013). Pharmacological brake-release of mRNA translation enhances cognitive memory. eLife.

[43] Dubey J, Ratnakaran N, Koushika SP (2015). Neurodegeneration and microtubule dynamics: Death by a thousand cuts. Front. Cell. Neurosci..

[44] Penazzi L, Bakota L, Brandt R (2016). Microtubule dynamics in neuronal development, plasticity, and neurodegeneration. Int. Rev. Cell Mol. Biol..

[45] Govek EE, Newey SE, Van Aelst L (2005). The role of the Rho GTPases in neuronal development. Genes Dev..

[46] Hoogenraad CC, Bradke F (2009). Control of neuronal polarity and plasticity—A renaissance for microtubules?. Trends Cell Biol..

[47] Heasman SJ, Ridley AJ (2008). Mammalian Rho GTPases: New insights into their functions from in vivo studies. Nat. Rev. Mol. Cell Biol..

[48] Vargas JP, Lopez JC, Portavella M (2009). What are the functions of fish brain pallium?. Brain Res. Bull..

[49] Portavella M, Vargas JP (2005). Emotional and spatial learning in goldfish is dependent on different telencephalic pallial systems. Eur. J. Neurosci..

[50] Fletcher ML (2012). Olfactory aversive conditioning alters olfactory bulb mitral/tufted cell glomerular odor responses. Front. Syst. Neurosci..

[51] Kass MD, Rosenthal MC, Pottackal J, McGann JP (2013). Fear learning enhances neural responses to threat-predictive sensory stimuli. Science.

[52] Laberge F, Roth G (2007). Organization of the sensory input to the telencephalon in the fire-bellied toad, *Bombina orientalis*. J. Comp. Neurol..

[53] Laberge F, Muhlenbrock-Lenter S, Dicke U, Roth G (2008). Thalamo-telencephalic pathways in the fire-bellied toad *Bombina orientalis*. J. Comp. Neurol..

[54] 54Nieuwenhuys, R., Donkelaar, H. J. t. & Nicholson, C. *The Central Nervous System of Vertebrates*. Vol. 3 (Springer, New York, 1998).

[55] Grossman EN, Giurumescu CA, Chisholm AD (2013). Mechanisms of ephrin receptor protein kinase-independent signaling in amphid axon guidance in *Caenorhabditis elegans*. Genetics.

[56] Borasio GD (1989). ras p21 protein promotes survival and fiber outgrowth of cultured embryonic neurons. Neuron.

[57] Sloniowski S, Ethell IM (2012). Looking forward to EphB signaling in synapses. Semin. Cell Dev. Biol..

[58] Cruz E (2015). Infralimbic EphB2 modulates fear extinction in adolescent rats. J. Neurosci..

[59] Mayr B, Montminy M (2001). Transcriptional regulation by the phosphorylation-dependent factor CREB. Nat. Rev. Mol. Cell Biol..

[60] Watkins JC, Evans RH (1981). Excitatory amino acid transmitters. Annu. Rev. Pharmacol. Toxicol..

[61] 61Garcia-Nafria, J., Herguedas, B., Watson, J. F. & Greger, I. H. The dynamic AMPA receptor extracellular region: A platform for synaptic protein interactions. *J. Physiol.* (2016). 10.1113/JP271844 PMC5043031 26891027

[62] Semenza GL, Wang GL (1992). A nuclear factor induced by hypoxia via de novo protein synthesis binds to the human erythropoietin gene enhancer at a site required for transcriptional activation. Mol. Cell Biol..

[63] Barrett TD (2011). Pharmacological characterization of 1-(5-chloro-6-(trifluoromethoxy)- 1H-benzoimidazol-2-yl)-1H-pyrazole-4-carboxylic acid (JNJ-42041935), a potent and selective hypoxia-inducible factor prolyl hydroxylase inhibitor. Mol. Pharmacol..

[64] Sugiyama M (2012). Homozygous and heterozygous GH transgenesis alters fatty acid composition and content in the liver of Amago salmon (*Oncorhynchus masou ishikawae*). Biol. Open.

[65] Kawamoto T (2003). Use of a new adhesive film for the preparation of multi-purpose fresh-frozen sections from hard tissues, whole-animals, insects and plants. Arch. Histol. Cytol..

[66] Komatsu Y, Kishigami S, Mishina Y (2014). In situ hybridization methods for mouse whole mounts and tissue sections with and without additional beta-galactosidase staining. Methods Mol. Biol. (Clifton, N.Y.).

[67] Mori T (2005). Genetic basis of phenotypic plasticity for predator-induced morphological defenses in anuran tadpole, Rana pirica, using cDNA subtraction and microarray analysis. Biochem. Biophys. Res. Commun..

