# Supplementary material for: Predation threats for a 24-h period activated the extension of axons in the brains of Xenopus tadpoles
Source: Sci Rep. 2020 Jul 16;10:11737. doi: 10.1038/s41598-020-67975-7 (PMC7367293; doi:10.1038/s41598-020-67975-7)
Supplement: Supplementary file 1 — Supplementary file1. [file 41598_2020_67975_MOESM1_ESM.pdf]

Supplementary Information

Predation threats for a 24-hour period activated the extension of axons in the brains of *Xenopus* tadpoles

Tsukasa Mori[1]*, Yoichiro Kitani[1, 2], Den Hatakeyama[1], Kazumasa Machida[1], Naoko Goto-Inoue[1], Satoshi Hayakawa[3], Naoyuki Yamamoto[4], Keiko Kashiwagi[5], Akihiko Kashiwagi[5]

Tsukasa Mori*,	Department of Marine Science and Resources, College of Bioresource Sciences, Nihon University
Yoichiro Kitani,	Institute of Nature and Environmental Technology Kanazawa University
Den Hatakeyama,	Department of Marine Science and Resources, College of Bioresource Sciences, Nihon University
Kazumasa Machdia,	Department of Marine Science and Resources, College of Bioresource Sciences, Nihon University
Naoko Goto-Inoue,	Department of Marine Science and Resources, College of Bioresource Sciences, Nihon University
Satoshi Hayakawa,	Department of Medicine, School of Medicine, Nihon University
Naoyuki Yamamoto,	Dept. of Animal Sciences, Graduate School of Bioagricultural Sciences, Nagoya University
Keiko Kashiwagi,	Amphibian Research Center (Building M), Hiroshima University
Akihiko Kashiwagi,	Amphibian Research Center (Building M), Hiroshima University

*Corresponding author: Tsukasa Mori
Department of Marine Science and Resources, Nihon University College of Bioresource Sciences, Kameino 1866, Fujisawa 252-0880, Japan
Tel. (81)-466-84-3682 Fax. (81)-466-84-3682

Table of Contents

Supplementary Table 1: All signal transduction pathways……………………………6
Supplementary Table 2: The top 10 up- and downregulated genes…………………..….6
Supplementary Table 3: Predicted diseases and functions……………………………..7
Supplementary Table 4: Actin cytoskeleton signaling network………………………14
Supplementary Table 5: The weight of the tadpoles used for predation threat……….18
Supplementary Table 6: The detail structures of adaptor and primer sequences …….19

Supplementary Table1

Canonical Pathway	6hr/cont	24hr/cont	10day/cont	5day-Out/cont
EIF2 Signaling	-3.211586168	-0.507092553	2.197401062	-3.887709572
Corticotropin Releasing Hormone Signaling	-3	2.5	-0.5	-3
Synaptic Long Term Potentiation	-2.857738033	1.459600898	-1.04257207	-3.544745039
GNRH Signaling	-2.857738033	1.876629727	-0.40824829	-3.265986324
CREB Signaling in Neurons	-2.523573073	2.064741605	-0.688247202	-2.828427125
HIPPO signaling	2.828427125	-1.414213562	1.414213562	2.121320344
Calcium Signaling	-2.357022604	2.357022604	-0.727606875	-2.182820625
Mitotic Roles of Polo-Like Kinase	-2.236067977	2.236067977	0.447213595	-2.236067977
Actin Cytoskeleton Signaling	-1.671258044	2.921186973	1.095445115	-1.460593487
Ephrin Receptor Signaling	-1.705605731	1.705605731	-1.04257207	-2.558408596
CDK5 Signaling	-2.523573073	-0.229415734	-1.605910137	-2.523573073
Role of NFAT in Cardiac Hypertrophy	-1.961161351	1.568929081	0.6	-2.745625892
Cardiac Hypertrophy Signaling	-1.566698904	2.263009527	0.87038828	-2.057983022
Protein Kinase A Signaling	-2.72165527	-1.360827635	-1.088662108	-1.483239697
NRF2-mediated Oxidative Stress Response	-1.414213562	2.357022604	-0.942809042	-1.885618083
ILK Signaling	1.961161351	2.501851166	0.577350269	1.347150628
Signaling by Rho Family GTPases	-0.538815906	3.535533906	1.414213562	-0.707106781
Ephrin B Signaling	-1.889822365	0.707106781	1.414213562	-2.121320344
Role of CHK Proteins in Cell Cycle Checkpoint Control	-1	2.333333333	-1	-1.666666667
Chemokine Signaling	-1.603567451	1.290994449	-0.774596669	-2.323790008
Dopamine-DARPP32 Feedback in cAMP Signaling	-1.527525232	1.963961012	-0.447213595	-1.963961012
Melatonin Signaling	-2.309401077	1.732050808	0	-1.732050808
ERK5 Signaling	-2.121320344	0.707106781	-0.707106781	-2.121320344
IL-1 Signaling	-1.154700538	2.110579412	0.577350269	-1.732050808
RhoA Signaling	-0.625543242	3.265986324	0.816496581	-0.816496581
P2Y Purigenic Receptor Signaling Pathway	-2.182820625	0.727606875	-0.242535625	-2.182820625
Agrin Interactions at Neuromuscular Junction	-1.897366596	0.632455532	-1.897366596	-0.632455532
PCP pathway	-0.904534034	1.732050808	0.577350269	-1.732050808
Androgen Signaling	-1.632993162	0.816496581	-0.816496581	-1.632993162
Hypoxia Signaling in the Cardiovascular System	0	1.632993162	0.816496581	-2.449489743
RhoGDI Signaling	0.784464541	-2.116950987	-1.347150628	0.577350269
VEGF Signaling	-1.507556723	1.507556723	0.301511345	-1.507556723
14-3-3-mediated Signaling	-2.110579412	0.301511345	-0.904534034	-1.507556723
Fcγ Receptor-mediated Phagocytosis in Macrophages and Monocytes	-0.942809042	1.885618083	-0.942809042	-0.942809042
B Cell Receptor Signaling	-0.39223227	1.568929081	0.6	-1.961161351
PI3K Signaling in B Lymphocytes	-0.942809042	0.942809042	0.727606875	-1.885618083

Amyloid Processing	−1.341640786	1.341640786	0.447213595	−1.341640786
Regulation of eIF4 and p70S6K Signaling	1.264911064	−1.897366596	0	1.264911064
FGF Signaling	−1.603567451	1.069044968	0	−1.603567451
IGF−1 Signaling	−0.904534034	−0.904534034	0.904534034	−1.507556723
Regulation of Actin−based Motility by Rho	−0.5	2.182820625	0.727606875	−0.727606875
Rac Signaling	−1	1	1	−1
Colorectal Cancer Metastasis Signaling	−0.755928946	0.755928946	0.928476691	−1.511857892
Gα12/13 Signaling	−0.942809042	0.688247202	1.147078669	−1.147078669
ERK/MAPK Signaling	−1.299867367	−1.133893419	0.185695338	−1.299867367
Huntington's Disease Signaling	0.25819889	−1.603567451	−1.069044968	0.774596669
Wnt/β−catenin Signaling	−0.894427191	0.894427191	0.894427191	−0.894427191
PDGF Signaling	−1.154700538	−0.904534034	0	−1.507556723
PPARα/RXRα Activation	0.471404521	−0.942809042	1.414213562	0.727606875
Integrin Signaling	−0.755928946	1.299867367	0.185695338	−1.299867367
Role of BRCA1 in DNA Damage Response	0	0.707106781	−0.707106781	−2.121320344
Remodeling of Epithelial Adherens Junctions	0.333333333	1.666666667	−0.333333333	−1
GM−CSF Signaling	−0.333333333	0.333333333	1	−1.666666667
Cell Cycle: G2/M DNA Damage Checkpoint Regulation	0.816496581	0	1.632993162	−0.816496581
Mouse Embryonic Stem Cell Pluripotency	−0.688247202	0.942809042	−0.688247202	−0.942809042
PPAR Signaling	0.832050294	1.386750491	0.277350098	0.577350269
PI3K/AKT Signaling	−0.894427191	−0.688247202	0.447213595	−0.942809042
fMLP Signaling in Neutrophils	−0.774596669	−0.25819889	−1.069044968	−0.774596669
mTOR Signaling	1.807392228	−0.5	0.5	0
Actin Nucleation by ARP−WASP Complex	−0.632455532	0.301511345	0.301511345	−1.507556723
Neuregulin Signaling	−1	−0.333333333	−1	−0.333333333
Neurotrophin/TRK Signaling	−0.904534034	0.301511345	−0.904534034	−0.301511345
STAT3 Pathway	−0.534522484	0.832050294	0	−0.832050294
AMPK Signaling	−0.242535625	−0.471404521	0.942809042	0
Role of Wnt/GSK−3β Signaling in the Pathogenesis of Influenza	−0.816496581	0	0	−0.816496581
p70S6K Signaling	−0.25819889	−0.25819889	−0.774596669	−0.25819889
ATM Signaling	0.301511345	0.301511345	0.301511345	−0.301511345
EGF Signaling	0	0	0.632455532	0
Lipid Antigen Presentation by CD1	N/A	N/A	N/A	N/A
Mechanisms of Viral Exit from Host Cells	N/A	N/A	N/A	N/A
Tight Junction Signaling	N/A	N/A	N/A	N/A
Ovarian Cancer Signaling	N/A	N/A	N/A	N/A
Branched−chain α−keto acid Dehydrogenase Complex	N/A	N/A	N/A	N/A
FLT3 Signaling in Hematopoietic Progenitor Cells	N/A	N/A	N/A	N/A
Sertoli Cell−Sertoli Cell Junction Signaling	N/A	N/A	N/A	N/A

HIF1α Signaling	N/A	N/A	N/A	N/A
FAK Signaling	N/A	N/A	N/A	N/A
Axonal Guidance Signaling	N/A	N/A	N/A	N/A
Acetyl-CoA Biosynthesis I (Pyruvate Dehydrogenase Complex)	N/A	N/A	N/A	N/A
Purine Nucleotides De Novo Biosynthesis II	N/A	N/A	N/A	N/A
TCA Cycle II (Eukaryotic)	N/A	N/A	N/A	N/A
CCR3 Signaling in Eosinophils	N/A	N/A	N/A	N/A
Role of MAPK Signaling in the Pathogenesis of Influenza	N/A	N/A	N/A	N/A
Xenobiotic Metabolism Signaling	N/A	N/A	N/A	N/A
Triacylglycerol Biosynthesis	N/A	N/A	N/A	N/A
DNA Double-Strand Break Repair by Non-Homologous End Joining	N/A	N/A	N/A	N/A
Glucocorticoid Receptor Signaling	N/A	N/A	N/A	N/A
RAN Signaling	N/A	N/A	N/A	N/A
Maturity Onset Diabetes of Young (MODY) Signaling	N/A	N/A	N/A	N/A
BMP signaling pathway	N/A	N/A	N/A	N/A
UDP-N-acetyl-D-galactosamine Biosynthesis II	N/A	N/A	N/A	N/A
Nucleotide Excision Repair Pathway	N/A	N/A	N/A	N/A
Hereditary Breast Cancer Signaling	N/A	N/A	N/A	N/A
Mitochondrial Dysfunction	N/A	N/A	N/A	N/A
Regulation of the Epithelial-Mesenchymal Transition Pathway	N/A	N/A	N/A	N/A
RAR Activation	N/A	N/A	N/A	N/A
Mismatch Repair in Eukaryotes	N/A	N/A	N/A	N/A
Molecular Mechanisms of Cancer	N/A	N/A	N/A	N/A
Regulation of IL-2 Expression in Activated and Anergic T Lymphocytes	N/A	N/A	N/A	N/A
2-ketoglutarate Dehydrogenase Complex	N/A	N/A	N/A	N/A
T Cell Receptor Signaling	N/A	N/A	N/A	N/A
Prostate Cancer Signaling	N/A	N/A	N/A	N/A
Glycolysis I	N/A	N/A	N/A	N/A
Epithelial Adherens Junction Signaling	N/A	N/A	N/A	N/A
Clathrin-mediated Endocytosis Signaling	N/A	N/A	N/A	N/A
Germ Cell-Sertoli Cell Junction Signaling	N/A	N/A	N/A	N/A
Inosine-5'-phosphate Biosynthesis II	N/A	N/A	N/A	N/A
TR/RXR Activation	N/A	N/A	N/A	N/A
Polyamine Regulation in Colon Cancer	N/A	N/A	N/A	N/A
UDP-N-acetyl-D-glucosamine Biosynthesis II	N/A	N/A	N/A	N/A
Estrogen Receptor Signaling	N/A	N/A	N/A	N/A
Cell Cycle Regulation by BTG Family Proteins	N/A	N/A	N/A	N/A
Human Embryonic Stem Cell Pluripotency	N/A	N/A	N/A	N/A
Breast Cancer Regulation by Stathmin1	N/A	N/A	N/A	N/A

Phenylalanine Degradation IV (Mammalian, via Side Chain)	N/A	N/A	N/A	N/A
Putrescine Degradation III	N/A	N/A	N/A	N/A
Role of Tissue Factor in Cancer	N/A	N/A	N/A	N/A
tRNA Charging	N/A	N/A	N/A	N/A
Unfolded protein response	N/A	N/A	N/A	N/A
Oxidative Phosphorylation	N/A	N/A	N/A	N/A
Gap Junction Signaling	N/A	N/A	N/A	N/A
Assembly of RNA Polymerase II Complex	N/A	N/A	N/A	N/A
Thyroid Cancer Signaling	N/A	N/A	N/A	N/A
Protein Ubiquitination Pathway	N/A	N/A	N/A	N/A
phagosome maturation	N/A	N/A	N/A	N/A

Supplementary Table 2

6hr			Association with human disease
ID	Symbol	Expr Log Ratio	
ENSDARG00000075513	CCDC136	9.746514321	Pervasive developmental disorder
ENSDARG00000044573	Cdc42	9.733015322	Noonan syndrome-like disorder
ENSDARG00000015201	PCMT1	9.670656249	Spina bifida, Huntington disease
ENSDARG00000017143	BRD9	8.382263758	Unknown
ENSDARG00000029524	IMPDH1	6.994353437	Retinitis pigmentosa
ENSDARG00000034063	GBP1	6.63420602	Systemic lupus erythematosus
ENSDARG00000031049	IGSF21	6.379378367	Unknown
ENSDARG00000061752	RIPOR2	6.129283017	Autosomal recessive deafness type 104
ENSDARG00000031886	IFT140	5.807354922	Retinitis pigmentosa
ENSDARG00000060152	FAM155B	5.247927513	Unknown
ENSDARG00000032126	SCG5	-9.388017285	Ecchymosis,hyperkeratosis
ENSDARG00000000161	ITSN2	-9.276124405	Nephrosis
ENSDARG00000063180	DOCK3	-8.451211112	Hereditary disorder
ENSDARG00000037030	CASZ1	-6.936637939	Dilated cardiomyopathy
ENSDARG00000057992	FST	-6.375039431	Cyanosis,hyperkeratosis
ENSDARG00000024746	HSP90AA1	-5.754887502	Metastatic non-small cell lung cancer
ENSDARG00000016375	ASNS	-4.491853096	Asparagine synthetase deficiency
ENSDARG00000024546	PLA2G4A	-4	Cytosolic phospholipase A2 alpha deficiency
ENSDARG00000100225	KIF16B	-3.169925001	Epithelial cancer in situ
ENSDARG00000063095	CTSF	-2.874469118	Kufs disease type B

10days			Association with human disease
ID	Symbol	Expr Log Ratio	
ENSDARG00000044573	Cdc42	9.283088353	Noonan syndrome-like disorder
ENSDARG00000017143	BRD9	7.431845787	Unknown
ENSDARG00000029524	IMPDH1	7.28077077	Retinitis pigmentosa
ENSDARG00000057987	UBA3	6.768184325	BRCA2 mutation negative breast cancer
ENSDARG00000100244	EBF3	6.549463819	Neurodevelopmental disorder
ENSDARG00000061752	RIPOR2	6.061776198	Autosomal recessive deafness type 104
ENSDARG00000068755	EXOSC8	5.914883386	Pontocerebellar hypoplasia type 1C
ENSDARG00000073801	KIAA2013	5.321928095	Unknown
ENSDARG00000087601	GPR153	5.189824559	Early-onset schizophrenia
ENSDARG00000060679	KDM1A	4.772589504	Intellectual disability,Kabuki syndrome
ENSDARG00000020596	MIGA1	-9.687375683	Unknown
ENSDARG00000041723	TUBB4B	-9.571752644	Metastatic breast cancer
ENSDARG00000032126	SCG5	-8.803054785	Ecchymosis,hyperkeratosis
ENSDARG00000074502	ZMIZ1	-7.751544059	Susceptibility to vitiligo
ENSDARG00000020387	NIPSNAP3A	-6.803054785	Unknown
ENSDARG00000026247	INSYN1	-6.562242424	Unknown
ENSDARG00000024746	HSP90AA1	-6.339850003	Metastatic non-small cell lung cancer
ENSDARG00000013414	LIN7A	-4.741466986	Unknown
ENSDARG00000038788	DNAI1	-3.770073906	Primary ciliary dyskinesia
ENSDARG00000060915	FBXL16	-3.459431619	Unknown

24hr			Association with human disease
ID	Symbol	Expr Log Ratio	
ENSDARG00000015201	PCMT1	10.02721489	Spina bifida, Huntington disease
ENSDARG00000044573	Cdc42	9.999295387	Noonan syndrome-like disorder
ENSDARG00000057987	UBA3	8.113742166	BRCA2 mutation negative breast cancer
ENSDARG00000017143	BRD9	7.297680549	Unknown
ENSDARG00000029524	IMPDH1	7.011227255	Retinitis pigmentosa
ENSDARG00000061752	RIPOR2	6.666756592	Autosomal recessive deafness type 104
ENSDARG00000031886	IFT140	6.163230349	Retinitis pigmentosa
ENSDARG00000087601	GPR153	5.988684687	Early-onset schizophrenia
ENSDARG00000073801	KIAA2013	5.984893108	Unknown
ENSDARG00000037188	RPA2	5.357552005	Unknown
ENSDARG00000033965	NUP58	-9.054378208	Unknown
ENSDARG00000026247	INSYN1	-7.562242424	Unknown
ENSDARG00000052094	NOTCH1	-7.430452552	Neoplasia, aortic valve disease
ENSDARG00000020387	NIPSNAP3A	-6.388017285	Unknown
ENSDARG00000037030	CASZ1	-6.351675438	Dilated cardiomyopathy
ENSDARG00000008740	ESF1	-6.273018494	Unknown
ENSDARG00000013414	LIN7A	-5.741466986	Unknown
ENSDARG00000024746	HSP90AA1	-5.339850003	Metastatic non-small cell lung cancer
ENSDARG00000016375	ASNS	-4.491853096	Asparagine synthetase deficiency
ENSDARG00000075536	CPNE1	-4.357552005	Atopic dermatitis

5days-Out			Association with human disease
ID	Symbol	Expr Log Ratio	
ENSDARG00000075513	CCDC136	9.471675214	Pervasive developmental disorder
ENSDARG00000020596	MIGA1	9.321928095	Unknown
ENSDARG00000015201	PCMT1	8.689997971	Spina bifida, Huntington disease
ENSDARG00000032126	SCG5	8.067882472	Hepatic steatosis, alopecia, ecchymosis
ENSDARG00000074502	ZMIZ1	7.8008999	Susceptibility to vitiligo
ENSDARG00000020387	NIPSNAP3A	7.090994532	Unknown
ENSDARG00000026247	INSYN1	6.577428828	Unknown
ENSDARG00000031886	IFT140	6.531381461	Retinitis pigmentosa
ENSDARG00000031049	IGSF21	5.437405312	Unknown
ENSDARG00000060915	FBXL16	5.426264755	Unknown
ENSDARG00000044573	Cdc42	-9.283088353	Noonan syndrome-like disorder
ENSDARG00000036038	HARBI1	-9.094077686	Unknown
ENSDARG00000017143	BRD9	-8.016808288	Unknown
ENSDARG00000079434	TMEM131L	-8.011227255	Productive infection by HIV-1
ENSDARG00000074777	ANK1	-7.511752654	Spherocytosis, hereditary spherocytosis
ENSDARG00000073801	KIAA2013	-5.906890596	Unknown
ENSDARG00000060679	KDM1A	-5.357552005	Intellectual disability,Kabuki syndrome
ENSDARG00000044776	CLIC3	-4.754887502	Rett syndrome
ENSDARG00000016375	ASNS	-4.426264755	Asparagine synthetase deficiency
ENSDARG00000060152	FAM155B	-4.222392421	Unknown

Supplementary Table3

Diseases and Bio Functions	Observation 1	Observation 2	Observation 3	Observation 4
Organismal death	6.21	−9.607	0.123	7.598
Viral Infection	−4.325	4.781	−0.073	−5.586
Morbidity or mortality	6.179	N/A	0.011	7.561
Congenital malformation of brain	1.867	−3.98	−2.177	3.809
Cell death of osteosarcoma cells	4.323	N/A	−1.604	5.516
Cell survival	−1.719	5.082	0.055	−4.194
Cell viability	−1.392	5.011	0.445	−3.929
Replication of Influenza A virus	−2.835	3.186	0.692	−3.61
Cell death	3.669	−1.57	1.41	3.425
Organization of cytoplasm	−2.601	4.109	−0.056	−3.171
Cell viability of tumor cell lines	−0.96	4.959	0.511	−3.463
Organization of cytoskeleton	−2.515	4.109	−0.056	−3.09
Congenital encephalopathy	1.867	−3.98	N/A	3.809
Transactivation	−2.807	3.244	0.227	−3.227
Replication of virus	−3.064	2.635	0.415	−3.302
Transactivation of RNA	−2.846	3.058	N/A	−3.263
Perinatal death	N/A	−5.198	0.277	3.434
Replication of RNA virus	−3.096	2.389	0.38	−3.02
Necrosis	3.093	−1.924	1.225	2.632
Infection of cells	−3.384	N/A	−0.011	−5.413
Dysgenesis	N/A	−4.79	N/A	3.885
Movement Disorders	2.861	−2.64	−0.653	2.475
Microtubule dynamics	−2	3.855	0.101	−2.557
Translation of RNA	2.382	N/A	−1.778	4.296
Cancer	1.501	−2.226	−2.112	2.589
Motor dysfunction or movement disorder	2.615	−2.524	−0.588	2.59
Infection by RNA virus	−3.022	N/A	0.178	−5.105
Infection by HIV−1	−2.771	N/A	N/A	−5.153
Transcription of RNA	−1.942	1.802	0.508	−3.317
Apoptosis	2.266	−1.436	2.172	1.576
Transcription	−2.152	1.6	0.42	−3.142
Liver lesion	1.491	−2.075	−0.834	2.406
Anemia	N/A	−2.983	−1.006	2.444
Genital tumor	−1.588	−2.376	−1.982	0.478
Malignant solid tumor	0.4	−1.83	−2.37	1.638
Development of malignant tumor	0.368	−2.441	−1.266	2.127

Development of body trunk	N/A	1.529	1.892	−2.634
Expression of protein	0.686	−2.037	−1.187	2.057
Cell proliferation of tumor cell lines	N/A	2.202	−1.013	−2.693
Cell death of cancer cells	2.533	N/A	−0.242	2.753
Cell cycle progression	−0.682	2.313	0.498	−1.918
Non−melanoma solid tumor	−0.524	−1.366	−1.882	1.531
Cell death of tumor cells	2.581	N/A	0.305	2.386
HIV infection	N/A	N/A	N/A	−5.234
Development of carcinoma	1.135	−1.887	−0.363	1.821
Incidence of tumor	−0.53	−2.696	−0.668	1.228
Gastrointestinal tract cancer	0.446	−2.382	N/A	2.179
Seizure disorder	2.82	N/A	N/A	2.127
Formation of cellular protrusions	N/A	2.742	0.322	−1.854
Transcription of DNA	−0.908	1.437	−0.061	−2.424
Formation of muscle	N/A	2.332	N/A	−2.486
Carcinoma	0.825	−1.416	−1.386	1.172
Tumorigenesis of epithelial neoplasm	0.549	−1.918	−0.668	1.648
Neonatal death	N/A	−4.267	0.485	N/A
Gastrointestinal tumor	−0.992	−2.062	−0.789	0.77
Progressive neurological disorder	−1.961	−0.218	N/A	−2.401
Aplasia or hypoplasia	N/A	N/A	−0.69	3.885
Genitourinary tumor	−2.001	−2.201	−0.009	−0.362
Growth Failure	N/A	N/A	N/A	4.563
Growth of organism	N/A	3.614	−0.926	N/A
Expression of mRNA	0.583	N/A	−1.434	2.499
Translation of protein	0.236	−1.65	−1.033	1.576
Digestive system cancer	1.079	−1.267	−0.963	1.108
Pelvic tumor	−1.715	−2.18	N/A	0.512
Development of adenocarcinoma	0.459	−2.279	−0.447	1.184
Development of digestive organ tumor	N/A	−1.753	−1.044	1.513
Translation of mRNA	N/A	N/A	N/A	4.296
Lymphocytic neoplasm	N/A	−1.863	−1.311	1.043
Nonhematologic malignant neoplasm	0.635	−1.32	−1.144	1.098
Infection of cervical cancer cell lines	N/A	N/A	0.55	−3.634
Cell death of tumor cell lines	1.925	N/A	1.584	0.665
Male genital neoplasm	−1.751	−1.432	N/A	−0.986
Expression of RNA	−1.329	0.768	0.118	−1.919
Frequency of tumor	−0.297	−2.13	−0.343	1.356
Bone marrow cancer	−1.564	1.149	N/A	−1.387

Lymphoproliferative disorder	N/A	−1.863	−1.224	1.005
Large intestine neoplasm	0.808	−1.974	N/A	1.192
Malignant genitourinary solid tumor	−1.093	−1.555	−0.747	0.527
Infection of tumor cell lines	N/A	N/A	0.073	−3.803
Development of central nervous system	−0.34	2.625	0.415	−0.475
Size of body	N/A	N/A	−0.134	−3.569
Tumorigenesis of tissue	−0.015	−0.983	−1.415	1.219
Renal lesion	N/A	−1.715	N/A	1.913
Abdominal neoplasm	−0.491	−1.645	−0.475	1.012
Cognitive impairment	1.463	0.038	−0.896	1.105
Lymphoid cancer	N/A	−1.629	−1.004	0.832
Lymphatic system tumor	N/A	−1.629	−1.004	0.832
Breast or colorectal cancer	−0.412	−1.664	N/A	1.373
Urinary tract tumor	N/A	−2	N/A	1.414
Abdominal cancer	0.481	−1.524	−0.655	0.723
Colorectal tumor	0.611	−1.852	N/A	0.918
Solid tumor	−0.618	−1.249	−0.943	0.533
Hematopoietic neoplasm	0.161	−0.805	−1.026	1.328
T−cell malignant neoplasm	N/A	−2.398	−0.896	N/A
Adenocarcinoma	0.541	−1.815	−0.478	0.371
Neoplasia of leukocytes	N/A	−1.863	−1.311	N/A
Lymphocytic cancer	N/A	−1.863	−1.311	N/A
Hematologic cancer of cells	N/A	−1.824	−1.311	N/A
Cardiogenesis	N/A	0.396	1.115	−1.569
Synthesis of protein	0.489	−0.323	−1.083	1.177
Lung carcinoma	N/A	−1.07	−0.555	1.442
Neural tube defect	N/A	−3.066	N/A	N/A
Liver tumor	1.093	−0.51	0.053	1.342
Translation	0.4	N/A	−0.894	1.693
Prostatic tumor	−1.961	N/A	N/A	−1.003
Hematologic cancer	N/A	−1.255	−0.894	0.808
Necrosis of tumor	2.581	N/A	0.305	N/A
Cell death of tumor	2.581	N/A	0.305	N/A
Extracranial solid tumor	−0.222	−1.626	−0.364	0.618
Acute leukemia	N/A	−1.095	−1.528	0.156
Cell death of malignant tumor	2.533	N/A	−0.242	N/A
Development of neurons	N/A	2.731	N/A	N/A
Neuromuscular disease	−1.13	N/A	N/A	−1.569
Development of body axis	−0.057	1.47	0.082	−1.079

Intestinal tumor	−0.093	−1.741	0.254	0.595
Non−hematological solid tumor	0.099	−0.961	−0.735	0.852
Gonadal tumor	N/A	−2.63	N/A	N/A
Congenital anomaly of digestive system	N/A	N/A	N/A	2.611
Development of head	N/A	1.649	−0.17	−0.76
Lymphohematopoietic neoplasia	N/A	−0.633	−0.773	1.154
Myeloid leukemia	N/A	1.149	N/A	−1.387
Intestinal cancer	1.121	N/A	N/A	1.412
Liver cancer	1.475	−0.317	−0.152	0.586
Neoplasia of cells	1.033	−1.246	N/A	−0.241
Liver carcinoma	1.513	−0.503	N/A	0.503
Head and neck tumor	−0.014	−0.966	1.165	0.331
Pericardial effusion	N/A	−1.912	−0.555	N/A
Myeloid neoplasm	−0.778	1.099	0.409	0.152
Bone marrow neoplasm	−0.778	1.099	0.409	0.152
Formation of actin filaments	N/A	N/A	−2.412	N/A
Activation of DNA endogenous promoter	−0.333	N/A	0.336	−1.728
Cancer of secretory structure	−0.109	−0.766	−0.686	−0.8
Ubiquitination of protein	N/A	N/A	2.345	N/A
Malignant neoplasm of large intestine	1	N/A	N/A	1.342
Lymphohematopoietic cancer	N/A	−1.064	−0.631	0.635
Interphase	N/A	0.8	−0.304	−1.168
Formation of filaments	N/A	N/A	−2.233	N/A
Liquid tumor	−0.639	−0.562	−0.702	0.306
Digestive organ tumor	−0.188	−0.978	−0.196	0.743
Cell death of breast cancer cell lines	N/A	N/A	2.073	N/A
Abdominal carcinoma	0.47	−0.78	−0.692	0.117
Lung adenocarcinoma	N/A	−0.785	N/A	1.23
Quantity of hematopoietic progenitor cells	N/A	N/A	−2.008	N/A
Progressive motor neuropathy	N/A	N/A	N/A	−2
Cell movement	N/A	1.994	N/A	N/A
Cancer of cells	N/A	−1.942	N/A	0.048
Hereditary myopathy	N/A	N/A	N/A	1.973
Lymphocytic leukemia	N/A	−1.969	N/A	N/A
Acute lymphoblastic leukemia	N/A	−1.969	N/A	N/A
Metabolism of protein	0.059	0.205	−0.812	0.877
Tumorigenesis of reproductive tract	N/A	−1.951	N/A	N/A
Gastrointestinal carcinoma	N/A	N/A	N/A	1.939
Ubiquitination	N/A	N/A	1.929	N/A

Fibrogenesis	N/A	N/A	−1.905	N/A
Non−small cell lung carcinoma	N/A	−0.415	N/A	1.488
Outgrowth of cells	N/A	1.902	N/A	N/A
Hepatobiliary system cancer	1.125	−0.317	−0.152	0.293
Seizures	N/A	N/A	N/A	1.879
Long−term potentiation	N/A	N/A	N/A	−1.862
Benign lesion	N/A	−1.838	N/A	N/A
Lymphoreticular neoplasm	N/A	−0.462	−0.182	1.177
Formation of brain	−0.246	0.955	−0.113	−0.433
Advanced malignant tumor	N/A	−1.313	−0.42	N/A
Primitive neuroectodermal tumor	N/A	N/A	N/A	−1.715
Quantity of neurons	N/A	N/A	N/A	−1.702
Renal tumor	N/A	N/A	N/A	1.633
Degeneration of embryoblast	N/A	N/A	1.633	N/A
Breast or pancreatic cancer	N/A	−0.365	−1.213	N/A
Pelvic cancer	N/A	−1.067	N/A	−0.479
Initiation of transcription	−1.545	N/A	N/A	N/A
Cell death of epithelial cell lines	N/A	N/A	1.539	N/A
Development of lung carcinoma	N/A	N/A	N/A	1.488
Quantity of vesicles	N/A	N/A	N/A	−1.488
Stress response of cells	N/A	N/A	−1.414	N/A
Development of sarcoma	N/A	−1.4	N/A	N/A
Genitourinary carcinoma	N/A	N/A	N/A	−1.387
Small GTPase mediated signal transduction	N/A	N/A	−1.387	N/A
Phosphorylation of protein	N/A	N/A	−1.336	N/A
Abdominal adenocarcinoma	−0.188	−0.84	N/A	−0.284
Proliferation of connective tissue cells	N/A	1.283	N/A	N/A
Secondary tumor	N/A	−1.085	−0.191	N/A
Morphogenesis of cardiovascular system	N/A	0.577	−0.686	N/A
Benign Tumors	N/A	−1.263	N/A	N/A
Development of lung tumor	N/A	−1.262	N/A	N/A
Growth of connective tissue	N/A	1.257	N/A	N/A
Cell death of epithelial cells	N/A	N/A	1.256	N/A
Advanced lung cancer	N/A	N/A	N/A	−1.219
Pancreatobiliary tumor	N/A	1.18	N/A	N/A
Epithelial neoplasm	N/A	N/A	N/A	1.165
Neurological signs	−0.284	N/A	−0.447	0.368
Development of genital tumor	N/A	−1.091	N/A	N/A
Quantity of lymphoid organ	N/A	N/A	−1.084	N/A

Breast or ovarian cancer	N/A	−1.067	N/A	N/A
Processing of RNA	N/A	0.469	N/A	−0.555
Necrosis of epithelial tissue	N/A	N/A	1.021	N/A
Colorectal cancer	N/A	N/A	N/A	1
Processing of mRNA	N/A	N/A	N/A	1
Quantity of synaptic vesicles	N/A	N/A	N/A	−1
Urinary tract cancer	N/A	N/A	N/A	1
Epilepsy	N/A	N/A	N/A	0.97
Leukemia	N/A	−0.45	N/A	−0.517
Apoptosis of epithelial cell lines	N/A	N/A	0.942	N/A
Ductal carcinoma	N/A	N/A	N/A	−0.928
Cell viability of breast cancer cell lines	N/A	N/A	−0.921	N/A
Cell death of kidney cell lines	N/A	N/A	0.854	N/A
Disorder of basal ganglia	N/A	N/A	N/A	−0.849
Embryonal tumor	N/A	N/A	N/A	−0.849
Prostate cancer	N/A	N/A	N/A	−0.842
Malignant neoplasm of male genital organ	N/A	N/A	N/A	−0.842
Genital tract cancer	N/A	N/A	N/A	−0.842
Hypoplasia of organ	N/A	N/A	−0.837	N/A
Cell death of embryonic cell lines	N/A	N/A	0.825	N/A
Dysplasia	0	−0.447	0	−0.378
Alveologenesis of lung	N/A	N/A	0.816	N/A
Thoracic neoplasm	N/A	−0.591	N/A	0.173
Splicing of RNA	N/A	N/A	N/A	0.762
Neoplasia of epithelial cells	N/A	0.735	N/A	N/A
Proliferation of embryonic cell lines	N/A	N/A	0.717	N/A
Hypoplasia	N/A	N/A	−0.69	N/A
Interphase of colorectal cancer cell lines	N/A	N/A	−0.655	N/A
Melanoma	N/A	0.651	N/A	N/A
Lung cancer	N/A	−0.262	−0.055	−0.321
Proliferation of fibroblast cell lines	N/A	N/A	N/A	−0.629
Thoracic cancer	N/A	−0.455	−0.055	0.077
Quantity of lymphoid tissue	N/A	N/A	−0.58	N/A
Respiratory system tumor	N/A	−0.431	N/A	−0.146
Lung tumor	N/A	−0.431	N/A	−0.146
Excision repair	N/A	N/A	N/A	0.572
Central nervous system solid tumor	N/A	0.57	N/A	N/A
Squamous−cell carcinoma	N/A	−0.557	N/A	N/A
Myeloproliferative neoplasm	N/A	0.53	N/A	N/A

Head and neck cancer	0.492	N/A	N/A	N/A
Extraadrenal retroperitoneal tumor	N/A	0.476	N/A	N/A
Sprouting	N/A	N/A	N/A	0.458
Mammary tumor	N/A	N/A	0.417	N/A
Senescence of cells	N/A	N/A	0.407	N/A
Brain lesion	N/A	−0.4	N/A	N/A
Viability	N/A	N/A	0.39	N/A
Chronic myeloproliferative neoplasm	N/A	0.379	N/A	N/A
Chronic myeloid leukemia	N/A	0.379	N/A	N/A
Organization of filaments	N/A	N/A	0.378	N/A
Organization of organelle	N/A	N/A	0.378	N/A
Proliferation of epithelial cell lines	N/A	N/A	0.357	N/A
Skin lesion	N/A	−0.338	N/A	N/A
Organization of actin cytoskeleton	N/A	N/A	−0.333	N/A
Hydrolysis of nucleotide	N/A	N/A	0.331	N/A
Skin tumor	N/A	−0.324	N/A	N/A
Sensitivity of cells	N/A	N/A	0.319	N/A
Catabolism of protein	N/A	N/A	N/A	−0.316
Nervous system neoplasm	N/A	0.314	N/A	N/A
Intracranial neoplasm	N/A	0.283	N/A	N/A
Brain tumor	N/A	0.283	N/A	N/A
Colon tumor	0.14	N/A	N/A	0.14
Repair of DNA	0.256	N/A	N/A	N/A
Cell death of kidney cells	N/A	N/A	0.247	N/A
Necrosis of kidney	N/A	N/A	0.242	N/A
Skin cancer	N/A	−0.239	N/A	N/A
Cell transformation	N/A	N/A	−0.231	N/A
T acute lymphoblastic leukemia	N/A	N/A	N/A	−0.218
T lymphoblastic leukemia/lymphoma	N/A	N/A	N/A	−0.218
Hepatocellular carcinoma	N/A	−0.199	N/A	N/A
Epithelial−mesenchymal transition of breast cell lines	N/A	N/A	0.173	N/A
Non−Hodgkin lymphoma	N/A	N/A	−0.152	N/A

Supplementary Table 4

	Supplementary Table.4		Exp Log Ratio				
Symbol	Entrez Gene Name	Ensembl(A1#)	(Ex6hr/Cont)	(Ex24hr/Cont)	(Ex10days/Cont)	(Ex5dayOut/Cont)	Location
ACTA2	actin, alpha 2, smooth muscle, aorta	ENSDARG00000036371	-0.669	2.966	-0.035	0.851	Cytoplasm
ACTC1	actin, alpha, cardiac muscle 1	ENSDARG00000042535	1.780	2.128	-1.755	3.265	Cytoplasm
ACTG1	21md.kazumasa	ENSDARG00000037746	-0.985	0.280	-0.314	-0.827	Cytoplasm
ACTN1	actinin, alpha 1	ENSDARG00000007219	-0.535	0.473	0.656	-1.128	Cytoplasm
ACTR2	ARP2 actin-related protein 2 homolog (yeast)	ENSDARG00000052438	0.241	0.348	0.933	-1.807	Plasma Membrane
APC	adenomatous polyposis coli	ENSDARG00000058868	-1.303	0.698	-0.068	-1.436	Nucleus
ARPC2	actin related protein 2/3 complex, subunit 2, 34kDa	ENSDARG00000075989	1.131	0.363	-0.144	0.212	Cytoplasm
ARPC3	actin related protein 2/3 complex, subunit 3, 21kDa	ENSDARG00000057882	0.135	-0.588	-1.811	2.024	Cytoplasm
EZR	ezrin	ENSDARG00000025091	-2.807	0.308	0.252	-0.252	Plasma Membrane
FGF8	fibroblast growth factor 8 (androgen-induced)	ENSDARG00000003399	-1.072	-0.241	-0.194	-0.662	Extracellular Space
FGF13	fibroblast growth factor 13	ENSDARG00000035056	-0.622	0.896	0.006	-0.965	Extracellular Space
HRAS	Harvey rat sarcoma viral oncogene homolog	ENSDARG00000098497	-1.029	-0.492	-0.757	-0.410	Plasma Membrane
LIMK2	LIM domain kinase 2	ENSDARG00000005104	1.722	0.004	-0.759	1.048	Cytoplasm
MAP2K1	mitogen-activated protein kinase kinase 1	ENSDARG00000007825	-0.363	0.603	0.290	-1.237	Cytoplasm
MYH4	myosin, heavy chain 4, skeletal muscle	ENSDARG00000067997	0.286	1.352	0.368	0.318	Cytoplasm
MYH11	myosin, heavy chain 11, smooth muscle	ENSDARG00000009782	1.579	0.619	0.195	0.463	Cytoplasm
MYL1	myosin, light chain 1, alkali; skeletal, fast	ENSDARG00000014196	0.117	2.287	-0.825	2.665	Cytoplasm
MYLPF	myosin light chain, phosphorylatable, fast skeletal muscle	ENSDARG00000053254	-1.296	2.548	0.276	-0.028	Cytoplasm
NCKAP1	NCK-associated protein 1	ENSDARG00000060853	-0.634	0.295	0.297	-1.182	Plasma Membrane
PAK2	p21 protein (Cdc42/Rac)-activated kinase 2	ENSDARG00000068177	-1.193	-1.356	0.209	-1.509	Cytoplasm
PFN2	profilin 2	ENSDARG00000012682	-0.270	0.579	0.182	-0.930	Cytoplasm
PIK3R4	phosphoinositide-3-kinase, regulatory subunit 4	ENSDARG00000060469	0.811	-0.123	0.462	0.131	Cytoplasm
PIP4K2A	phosphatidylinositol-5-phosphate 4-kinase, type II, alpha	ENSDARG00000003776	-0.532	0.352	0.272	-0.791	Cytoplasm
PIP4K2C	phosphatidylinositol-5-phosphate 4-kinase, type II, gamma	ENSDARG00000031020	-0.778	0.874	0.939	-1.524	Cytoplasm
RDX	radixin	ENSDARG00000028740	0.302	0.218	0.136	0.269	Cytoplasm
RHOA	ras homolog family member A	ENSDARG00000094673	0.000	-0.206	0.485	-0.807	Cytoplasm
ROCK1	Rho-associated, coiled-coil containing protein kinase 1	ENSDARG00000058993	-1.379	0.054	0.547	-2.248	Cytoplasm
ROCK2	Rho-associated, coiled-coil containing protein kinase 2	ENSDARG00000017500	-0.059	1.566	1.642	-1.826	Cytoplasm
RRAS2	related RAS viral (r-ras) oncogene homolog 2	ENSDARG00000036252	-1.000	-0.333	-1.430	-0.389	Plasma Membrane
TLN2	talin 2	ENSDARG00000017901	-0.263	0.830	-0.082	-0.766	Nucleus
TTN	titin	ENSDARG00000028213	2.000	4.585	2.585	0.737	Cytoplasm
VAV2	vav 2 guanine nucleotide exchange factor	ENSDARG00000044718	-1.201	0.465	0.731	-2.660	Cytoplasm
WAS	Wiskott-Aldrich syndrome	ENSDARG00000015149	-1.637	0.402	0.382	-1.061	Cytoplasm
WASF1	WAS protein family, member 1	ENSDARG00000060349	-0.303	-0.407	-0.870	0.410	Nucleus

Supplementary table 5

Ex10day		Cont		Ex 5day-Out		Ex 24hr		Ex 6hr	
No.	Weight(g)	No.	Weight(g)	No.	Weight(g)	No.	Weight(g)	No.	Weight(g)
1	0.084	1	0.068	1	0.075	1	0.077	1	0.054
2	0.086	2	0.070	2	0.083	2	0.085	2	0.060
3	0.088	3	0.079	3	0.091	3	0.088	3	0.063
4	0.089	4	0.079	4	0.094	4	0.088	4	0.065
5	0.093	5	0.080	5	0.098	5	0.089	5	0.069
6	0.094	6	0.085	6	0.101	6	0.094	6	0.071
7	0.096	7	0.086	7	0.101	7	0.103	7	0.074
8	0.099	8	0.086	8	0.102	8	0.106	8	0.075
9	0.101	9	0.090	9	0.103	9	0.112	9	0.086
10	0.105	10	0.090	10	0.105	10	0.120	10	0.089
11	0.109	11	0.091	11	0.105	11	0.122	11	0.091
12	0.109	12	0.091	12	0.106	12	0.124	12	0.098
13	0.109	13	0.091	13	0.110	13	0.125	13	0.099
14	0.109	14	0.091	14	0.116	14	0.125	14	0.101
15	0.113	15	0.093	15	0.118	15	0.125	15	0.103
16	0.117	16	0.094	16	0.119	16	0.129	16	0.107
17	0.120	17	0.095	17	0.124	17	0.133	17	0.113
18	0.122	18	0.096	18	0.124	18	0.134	18	0.115
19	0.122	19	0.097	19	0.125	19	0.136	19	0.115
20	0.123	20	0.103	20	0.129	20	0.137	20	0.117
21	0.123	21	0.105	21	0.129	21	0.137	21	0.117
22	0.126	22	0.107	22	0.131	22	0.137	22	0.117
23	0.132	23	0.108	23	0.132	23	0.138	23	0.117
24	0.132	24	0.109	24	0.133	24	0.144	24	0.120
25	0.133	25	0.109	25	0.133	25	0.145	25	0.123
26	0.133	26	0.112	26	0.136	26	0.147	26	0.124
27	0.136	27	0.113	27	0.136	27	0.147	27	0.129
28	0.138	28	0.115	28	0.143	28	0.151	28	0.132
29	0.139	29	0.116	29	0.145	29	0.151	29	0.133
30	0.143	30	0.119	30	0.146	30	0.152	30	0.133
31	0.144	31	0.120	31	0.146	31	0.153	31	0.133
32	0.145	32	0.122	32	0.147	32	0.153	32	0.134
33	0.145	33	0.123	33	0.147	33	0.154	33	0.134
34	0.146	34	0.124	34	0.153	34	0.154	34	0.136
35	0.147	35	0.124	35	0.159	35	0.157	35	0.140
36	0.147	36	0.127	36	0.161	36	0.158	36	0.142
37	0.148	37	0.128	37	0.161	37	0.163	37	0.145
38	0.150	38	0.129	38	0.163	38	0.163	38	0.146
39	0.152	39	0.130	39	0.164	39	0.164	39	0.147
40	0.158	40	0.133	40	0.166	40	0.168	40	0.149
41	0.165	41	0.134	41	0.169	41	0.173	41	0.152
42	0.166	42	0.137	42	0.182	42	0.188	42	0.153
43	0.168	43	0.138	43	0.193	43	0.188	43	0.156
44	0.172	44	0.144	44	0.193	44	0.207	44	0.158
45	0.177	45	0.152	45	0.194	45	0.224	45	0.163
46	0.177	46	0.159	46	0.195	46	0.230	46	0.173
47	0.184	47	0.177	47	0.200	47	0.231	47	0.188
48	0.194	48	0.187	48	0.204	48	0.236	48	0.203
49	0.207	49	0.204	49	0.211	49	0.236	49	0.221
50	0.283	50		50	0.240	50	0.237	50	0.239
Average	0.136	Average	0.113	Average	0.141	Average	0.149	Average	0.124
Std Dev	0.036	Std Dev	0.029	Std Dev	0.037	Std Dev	0.041	Std Dev	0.040
SEM	0.005	SEM	0.004	SEM	0.005	SEM	0.006	SEM	0.006

Supplementary Table 6

Oligo(dT)_EcoP15I_adapter_primer（Length: 53）
biotin – 5' – CTG ATC TAG AGG TAC CGG ATC CCA GCA G(T) 25 –3

adapter 1: 5'_NlaIII–EcoP_adapter–1Length: 43）
5'–ACA CTC TTT CCC TAC ACG ACG CTC TTC CGA TCT CAG CAG CAT G–3'

adapter 1: 5'_NlaIII–EcoP_adapter–1（Length: 39）
5'–/5Phos/ CTG CTG AGA TCG GAA GAG CGT CGT GTA GGG AAA GAG TGT/3AmMC7/–3'

adapter 2: 3'_NlaIII–EcoP_adapter2（Length: 34）
5'–GTG ACT GGA GTT CAG ACG TGT GCT CTT CCG ATC T–3'

adapter 2: 3'_NlaIII–EcoP_adapter2（Length: 34）
5'–/5Phos/ AGA TCG GAA GAG CAC ACG TCT GAA CTC CAG TCA C/3AmMC7/–3'

Universal PCR Primer for illumina
5'–AATGATACGGCGACCACCGAGATCTACACTCTTTCCCTACACGACGCTCTTCCGATCT–3'

NEBNext Index1 Primer for Illumina

5´–CAAGCAGAAGACGGCATACGAGATCGTGATGTGACTGGAGTTCAGACGTGTGCTCTTC
CGATC–s–T–3´

Where –s– indicates phosphorothioate bond
